# Populations of the Minor α-Conformation in AcGXGNH_2_ and the α-Helical Nucleation Propensities

**DOI:** 10.1038/srep27197

**Published:** 2016-06-03

**Authors:** Yanjun Zhou, Liu He, Wenwen Zhang, Jingjing Hu, Zhengshuang Shi

**Affiliations:** 1 School of Chemistry and Chemical Engineering, Huazhong University of Science and Technology, 1037 Luoyu Road, Wuhan 430074, P.R. China

## Abstract

Intrinsic backbone conformational preferences of different amino acids are important for understanding the local structure of unfolded protein chains. Recent evidence suggests α-structure is relatively minor among three major backbone conformations for unfolded proteins. The α-helices are the dominant structures in many proteins. For these proteins, how could the α-structures occur from the least in unfolded to the most in folded states? Populations of the minor α-conformation in model peptides provide vital information. Reliable determination of populations of the α-conformers in these peptides that exist in multiple equilibriums of different conformations remains a challenge. Combined analyses on data from AcGXPNH_2_ and AcGXGNH_2_ peptides allow us to derive the populations of PII, β and α in AcGXGNH_2_. Our results show that on average residue X in AcGXGNH_2_ adopt PII, β, and α 44.7%, 44.5% and 10.8% of time, respectively. The contents of α-conformations for different amino acids define an α-helix nucleation propensity scale. With derived PII, β and α-contents, we can construct a free energy-conformation diagram on each AcGXGNH_2_ in aqueous solution for the three major backbone conformations. Our results would have broad implications on early-stage events of protein folding.

Protein sequence-structure relationships are of fundamental importance to the field of protein physical chemistry^1^,^2^,^3^,^4^. Intrinsic backbone conformational preferences of 20 amino acids determine the local structure of unfolded protein chains; these intrinsic preferences might guide the folding processes at early stages of protein folding. From this respect, the intrinsic backbone conformational preferences of different amino acids are part of the "folding mechanism" that remains poorly understood after more than 50 years since the protein folding question was raised^1^,^2^,^3^,^4^. Currently, to predict protein structure from amino acid sequences, database-based strategies are more successful than the physics-based algorithms. Advances in the physics-based algorithms demand continuous improvements in force field accuracy. The intrinsic backbone conformational preference data are crucial for this purpose.

Among three major backbone conformations, α-structure is relatively minor compared to polyproline II (PII) and β-conformations in model unfolded peptides as demonstrated by recent lines of independent evidence^5^,^6^,^7^,^8^,^9^,^10^,^11^,^12^,^13^,^14^,^15^,^16^,^17^,^18^,^19^,^20^,^21^,^22^,^23^,^24^,^25^,^26^,^27^,^28^,^29^. Reliable derivation of populations of the minor α-conformers in model peptides that exist in multiple equilibriums of different backbone conformations remains a challenge^11^,^18^,^19^,^23^,^24^,^25^,^27^. NMR measurements can only be carried out on a slow time scale as compared to backbone conformers’ lifetimes which lie in the range of 10–200 ps, conformational averaging over different conformers occurs during NMR measurements. The optical spectroscopy results are measured on a fast time scale and various optical spectra can be used to detect different backbone conformations^6^,^8^,^9^,^16^,^17^,^23^. However, most optical techniques suffer from their resolutions: band overlapping in the measured spectra generally cause significant uncertainties during quantitative analysis, particularly for accurate derivations of minor conformers. In our previous work, quantitative account of the sampled conformation in AcGGXGGNH_2_ and XAO by NMR ^3^J(H_α_-H_N_) (^3^J_αN_) coupling constants was carried out through a two-state analysis for an equilibrium mainly between PII and β conformations; α-population was ignored completely as an approximation^11^,^18^. Here we have designed two series of peptides: AcGXPNH_2_ and AcGXGNH_2_ (X ≠ Gly, Pro). Combined analyses on data from both series allow us to derive the populations of three major conformers including PII, β and α in AcGXGNH_2_.

Proline is unique among the amino acids in that it has a five-membered ring which has a dramatic effect on the conformational preferences of the preceding residue. In AcGXPNH_2_ peptides, X can only adopt PII or β conformations as steric clashes between the C^δ^ of proline and both the C^β^ and amide nitrogen of residue X make α-conformation inaccessible to residue X^30^,^31^,^32^. With the measured ^3^J_αN_ coupling constants of X, previous procedure through a two-state analysis for the equilibrium between PII and β is justified for AcGXPNH_2_ peptides^11^,^18^. PII to β population ratio for each of AcGXPNH_2_ can be determined; assuming the ratio for X in AcGXPNH_2_ and AcGXGNH_2_ is approximately the same, we can derive the population of α-conformer in AcGXGNH_2_ peptides through equation (1), see Supplementary Information for derivation of the equation in which x_α_(GXG) denotes the percentage of α-conformer in AcGXGNH_2_; ^3^J_αN_(GXP) and ^3^J_αN_(GXG), measured ^3^J_αN_ coupling constants of X in AcGXPNH_2_ and AcGXGNH_2_; ^3^J_αN_(α), standard ^3^J_αN_ coupling constant of a residue in α-helices.


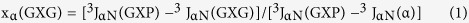


Further, we can derive the populations of PII and β in AcGXGNH_2_ through equations (2) and (3) in which ^3^J_αN_(PII),^3^J_αN_(β) and ^3^J_αN_(α) denote standard ^3^J_αN_ coupling constant of a residue in PII, β-, and α-conformations, respectively; x_PII_(GXG), x_β_(GXG) and x_α_(GXG) denote the percentage of PII, β- and α-conformations in AcGXGNH_2_, respectively. With the derived percentage values, the free energy-conformation diagrams of AcGXGNH_2_ in aqueous solution can be constructed for the three major backbone conformations.


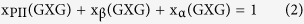



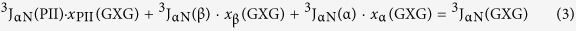


Derived results show that on average residue X in AcGXGNH_2_ adopt PII, β, and α 44.7%, 44.5% and 10.8% of time, respectively. Importantly, minor populated α-conformations of different amino acids in AcGXGNH_2_ determine their varying α-helix nucleation capabilities^33^. According to Zimm-Bragg theory^34^, helix-to-coil transition can be described by a nucleation constant σ and helix propagation constants s, the product σ∙s represents the probability of formation of an α-helical segment comprising three residues^34^,^35^,^36^. From our derived vales of x_α_, we can estimate the probability for Ala peptides, σ∙s = (x_α_)^3^ = 4.29 × 10^−3^ (x_α_ = 0.1625 for Ala), this value is very close to those reported^37^. Our free energy-conformation diagrams would set a foundation for physics-based algorithmic developments for protein structure predictions^38^,^39^.

## Results and Discussions

### Model peptides AcGXPNH_2_ and AcGXGNH_2_ and their CD spectra

Our previous study on AcGGXGGNH_2_ peptides showed that these peptides are present predominantly in the extended PII or β structure, around 10% α or turn structures could be present, but the exact percentage of α or turn conformation could not be determined. In AcGXGNH_2_, X is expected to sample all three major backbone conformations, with PII or β structure being dominant and α basin being minor; in AcGXPNH_2_, however, X can sample only PII or β conformations. To avoid end and charge effects, two peptide series of this study have both ends blocked^27^. CD spectra for most AcGXGNH_2_ peptides except those with ring side chains (His, Trp, Tyr, Phe) show the characteristic far-UV CD signature of a mixture of PII and β conformations, with a strong negative band at ≈198 nm and a weak positive band or shoulder at ≈215 nm^18^,^28^,^40^(Fig. S1). CD spectra of AcGXGNH_2_ are very similar to those of AcGGXGGNH_2_^18^. CD spectra of AcGXPNH_2_ are obscured by the contributions from Pro (Fig. S1). Small populations of Pro could exist in *cis* configurations; typical CD spectra of Pro peptides in PII helix usually shift to a longer wavelength as compared to those of non-Pro peptides. As a result, interpretation of CD spectra for AcGXPNH_2_ is not very obvious. Differential spectra between AcGXPNH_2_ and AcGXGNH_2_ reveal that Pro exists as a mixture of PII and PI (polyproline I) helices in AcGXPNH_2_^41^; thus CD spectra of AcGXPNH_2_ reflect contributions from both X and Pro, contributions from X are expected to show the characteristic far-UV CD signature of a mixture of PII and β conformations, similar to those observed for AcGXGNH_2_.

### Contents of α-conformers in AcGXGNH_2_ correlate with α-helix nucleation capabilities of X

**Figure 1:**
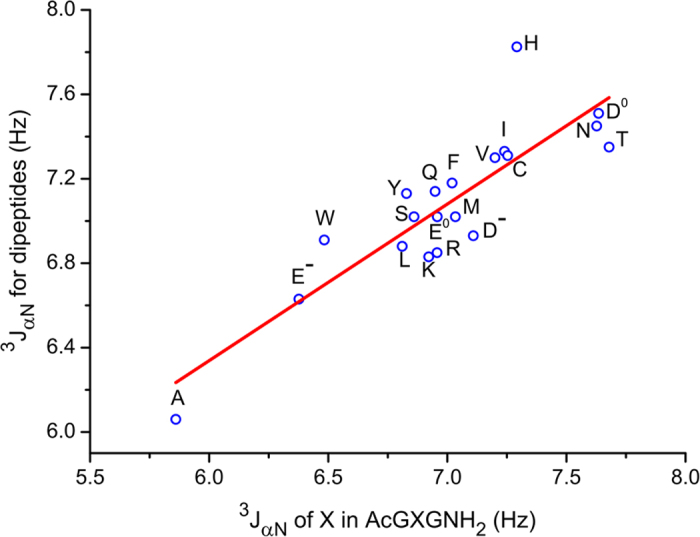
The ^3^J_αN_ coupling constants measured for AcGXGNH_2_ peptides are plotted against those for amino acid dipeptides.

^3^J_αN_ coupling constant is directly related to the backbone ϕ angle by Karplus equations^42^,^43^. Measured ^3^J_αN_ values at 25 °C (pH = 4.0) for AcGXGNH_2_ and AcGXPNH_2_ peptides are shown in Table 1 (see Fig. S2 for the NMR spectra and results of fitting). In AcGXPNH_2_, there is a slow *trans*-to-*cis* equilibrium for Pro, ^3^J_αN_ for both *cis*- and *trans*- species are well resolved in 1D ^1^H NMR spectra, here only ^3^J_αN_ values of X corresponding to *trans*-Pro are reported. Measured ^3^J_αN_ coupling constants for AcGXGNH_2_ are compared to those for dipeptides (blocked amino acids)^19^ at 30 °C (pH = 4.9) in Fig. 1. The plot reveals a good agreement between two sets of coupling constants (R = 0.86).

^3^J_αN_ values for AcGXGNH_2_ are smaller than those for AcGXPNH_2_ for most amino acids except for residues Asp (pH = 2.0 and 6.0), Asn and Thr. Excluding Thr, Asn and Asp’s, ^3^J_αN_ values for AcGXGNH_2_ are on average 0.41 Hz smaller than those for AcGXPNH_2_. The smaller ^3^J_αN_ values for AcGXGNH_2_ are consistent to X samples all three major backbone conformations in AcGXGNH_2_, while X samples only PII and β conformations in AcGXPNH_2_ (Thr, Asn and Asp are excluded). For AcGXPNH_2_ (X = Thr, Asn and Asp), X is expected to form turn structures^44^; it explains smaller observed ^3^J_αN_ values for these residues in AcGXPNH_2_ compared to those in AcGXGNH_2_. For all other amino acids, contents of α conformations in AcGXGNH_2_ can be calculated from equation (1), in which ^3^J_αN_(α) is assigned to be 4.11 Hz, corresponding to a ϕ value of −60° (Table 1). For Thr, Asn and Asp (pH = 2.0 and 6.0) in AcGXGNH_2_, their contents of α conformations cannot be determined. It is a conservative and proximate practice to assign the values to be 0.04, 0.025, 0.02 and 0.05 for Thr, Asn and Asp (pH = 2.0 and 6.0), respectively, corresponding to the values from dipeptides by Grdadolnik *et al.*^23^ (Table 1). Contents of α conformations derived from blocked amino acids are significantly smaller than our values, 5.2 % *vs*. 12.6 % on average with Thr, Asn and Asp being excluded.

**Figure 2:**
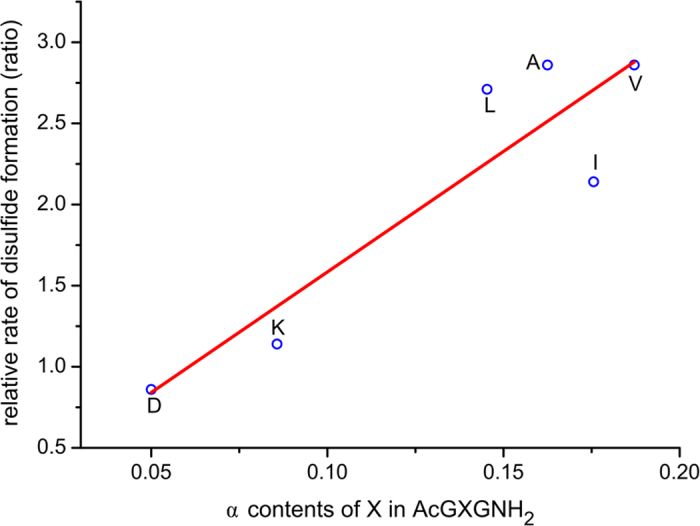
The correlation of determined α-contents for AcGXGNH_2_ and the relative rates of disulfide formation in a synthetic model.

Our results indicate that x_α_ values for hydrophobic or aromatic amino acids are significantly larger than those for polar amino acids, 14.9% vs. 7.4% on average. The differences among different non-polar residues are marginal (Table 1). Contents of minor populated α-conformations of different amino acids in AcGXGNH_2_ determine their varying α-helix nucleation propensities. Our results suggest that: for non-polar amino acids, the nature or the size of side chains, being aromatic ring or β-branching, do not have strong steric impact on helix nucleation, in contrast to their strong effects on helix propagation due to different steric constraints. The x_α_ values observed show no correlation to any α-helix propensity scales^45^,^46^,^47^ that report mainly the propensity of amino acid residues to propagate on a preformed helix; the observation corroborates the conclusion by Miller *et al.*^33^ Effects of individual side chains on helix nucleation are difficult to deconvolute from those of helix propagation. Recently, Miller *et al.* have successfully separated the effects through studying a synthetic model and found that amino acid side chains contribute in a completely different manner to nucleation than to propagation^33^. In this study, the relative rates of disulfide formation serve as indirect indicators for different residues’ α-helix nucleation capabilities. Our derived populations of α conformations in AcGXGNH_2_ are compared to the relative rates of disulfide formation for limited amino acids by Miller *et al.*^33^ (Table S1); a good correlation is revealed (Fig. 2, R = 0.88).

From derived vales of x_α_, we can calculate the probability of forming an α-helical segment comprising three residues, σ∙s = (x_α_)^3^ = 1.26 × 10^−3^ if we use the average value of x_α_ for all amino acids. For Ala peptides, we can determine the probability, σ∙s = (x_α_)^3^ = 4.29 × 10^−3^ (x_α_ = 0.1625 for Ala). The value is very close to those reported for Ala-rich peptides (the measured σ = 0.004 ± 0.002 with s_Ala_ = 1.4–1.6)^37^. As parameters, products of (x_α1_ • x_α2_ • x_α3_) for a combination of three different amino acids would be sensitive indicators to uncover the potential helix nucleation sites within sequences that form α-helices. From the derived x_α_ values (Table 1), we predict sequences comprised of Val, Trp, Ile, His, Glu (pH = 2.0) and Ala are most likely the nucleation sites at early stages of α-helix formation; whereas sequences comprised of Asp, Cys, Asn and Thr (Pro and Gly are not considered here) are least likely the nucleation sites. Fast folding kinetic studies on model protein/peptides are expected to validate or invalidate our predictions.

### Contents of PII and β conformations in AcGXGH_2_ and construction of free energy-conformation diagrams for three major backbone conformations

Contents of PII and β conformations in AcGXGNH_2_ can be calculated using equations (2) and (3) (Table 1). We assign standard ^3^J_αN_ values for PII and β conformations to be 5.42 and 9.30 Hz, respectively. The value of 5.42 Hz for ^3^J_αN_(PII) corresponds to a ϕ value of −70°; the value of 9.30 Hz for ^3^J_αN_(β) is the result from fitting measured ^3^J_αN_ values on blocked dipeptides to their β-populations derived from optical spectroscopic bands^23^. X in AcGXGNH_2_ adopts predominantly the extended PII or β conformations; on average, X samples about the same amount of time in PII or β basin, 44.7% *vs*. 44.5%. Our analysis indicates that β-contents or ΔG values for corresponding PII to β equilibriums show weak or reasonable correlations with β propensity scales (weak with β-contents and reasonable with ΔG), consistent to the observation in AcGGXGGNH_2_ peptides^18^. Correlations between ΔG and the β-sheet scale by Kim and Berg^48^ are shown in Fig. S3.

**Figure 3:**
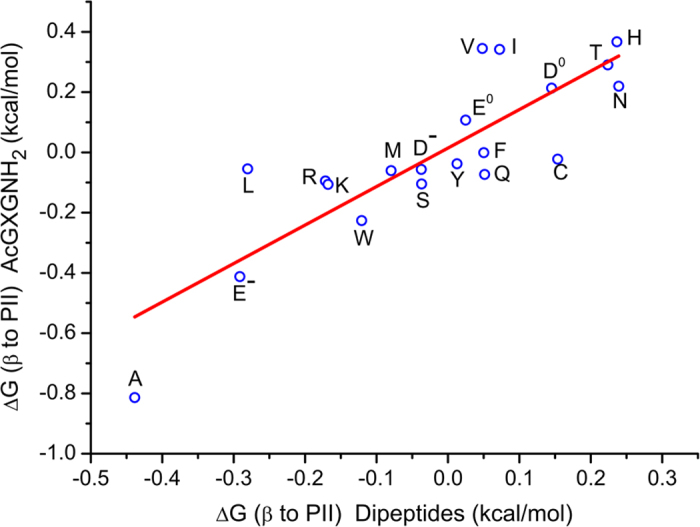
The correlation of ΔG(β to PII) derived for AcGXGNH_2_ and that for dipeptides.

A more relevant comparison is between our data to those from blocked amino acids (dipeptides). Grdadolnik *et al.* have determined populations of the three major backbone conformations in 19 amino acid dipeptides (N-acetyl-X-N′-methylamide) by using the amide III region of the peptide infrared and Raman spectra^23^. The work by Grdadolnik *et al.* represents a major advance in band assignments of the peptide infrared and Raman spectra to different backbone conformations^23^. This advance made determination of backbone conformational distribution possible. If we compare our derived ΔG values for PII to β transitions to those derived for dipeptides, we find a reasonably strong correlation (Fig. 3, R = 0.84). Comparison of this correlation to the one in Fig. 1 (R = 0.86) indicates that the correlation between ΔG values is limited to that between ^3^J_αN_ values. Given totally independent strategies on different systems were used, the correlation provides validations for both methods.

The average length of β-strands in β-sheets is about 6 residues, the probabilities of forming a β-strand of 6 residues is (x_β_)^6^ = 7.77 × 10^−3^ if we use the average value of x_β_ for all amino acids. Considering strands of 3–6 amino acids long might all play important roles in the early stages of β-hairpin folding, the population of a preformed β-strand of 3 residues long would reach as high as 20% (corresponding to x_β_ = 0.585). Following the procedure for α-helices, products of (x_β1_ • x_β2_ • x_β3_) for a combination of three different amino acids might be used to locate the potential sites that form β-strands at early stages of protein folding. Similarly, from the derived x_β_ values (Table 1), we predict sequences comprised of Thr, Asp (pH = 2), Asn, His, Ile and Val are most likely the sites that tend to form nascent β-strands; whereas sequences comprised of Ala, Glu (pH = 6) and Trp (Pro and Gly are not considered here) are least likely the sites to form nascent β-strands. Nascent β-strands then initiate a productive or non-productive collision.

**Figure 4:**
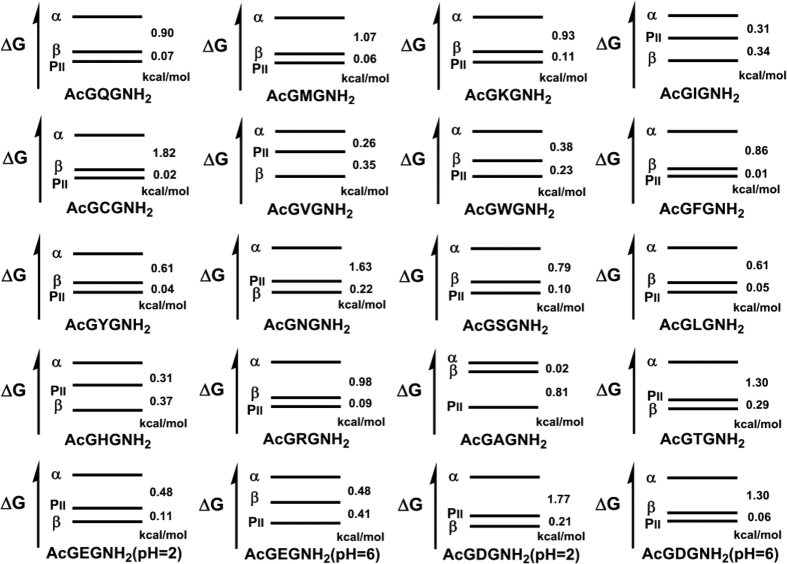
Derived free energy-conformation diagrams for AcGXGNH_2_.

With the derived PII, β and α-contents, we can construct a free energy-conformation diagram on each AcGXGNH_2_ in aqueous solution for the three major backbone conformations (Fig. 4). The diagrams clearly show that the free energy level for α-basin is the highest among three for all amino acids; the free energy level for PII basin is the lowest for most amino acids except for Ile, Val, Asn, His, Thr, Glu (pH = 2.0) and Asp (pH = 2.0). Together with the results on 19 amino acid dipeptides from the optical spectroscopic data^23^, it is our believe that the derived free energy-conformation diagrams would provide a bench mark for testing predicting calculations of conformational energy maps of flexible model peptides^38^,^39^.

### Turn conformations in AcGNPNH_2_, AcGTPNH_2_ and AcGDPNH_2_ (pH = 2 and 6) and effects of different ^3^J_αN_(PII) and ^3^J_αN_(β) values on data analysis

We have detected significant turn structures in AcGNPNH_2_, AcGTPNH_2_ and AcGDPNH_2_ (pH = 2 and 6) as shown in Table 2. This observation is consistent with the findings by Hagarman *et al.*^44^ In this study, we assign standard ^3^J_αN_ values for PII and β conformations to be 5.42 and 9.30 Hz, respectively. In our previous study on AcGGXGGNH_2_ peptides, a set of residue-specific ^3^J_αN_ reference values for PII and β conformations were used^18^,^49^ (See Table 1 of reference 18). If we use the previous set of reference values to analyze the data in this study, slightly different PII, β and α-contents are obtained. Comparison of two sets of results indicates they are matched to each other overall with derived conclusions being the same. (See Supplementary Information for details). Regardless, the choice of different ^3^J_αN_(PII) and ^3^J_αN_(β) values has no effects on our derived x_α_ values for X in AcGXGNH_2_ as implied by equation (1) (see Supplementary Information for derivation of the equation).

### NOE data and error analysis

NOEs can be used to analyze the conformations. Amide region of NOESY spectra for AcGXGNH_2_ peptides are shown in Fig. S10. Strong d_αN_(i, i + 1) NOE cross peaks are observed for X residues in AcGXGNH_2_ peptides, while the intensities of d_αN_(i, i) NOEs are weakened by about two- to fourfold relative to those of d_αN_(i, i + 1) NOEs; the d_NN_(i, i + 1) NOEs are not measurable due to their weak intensities and being very close to the diagonal peaks. These results indicate that AcGXGNH_2_ peptides are present predominantly in the extended PII or β-conformations that are consistent with our conclusion through analyzing coupling constant data. Figure S2 shows the amide region of 1D NMR spectra for all AcGXPNH_2_ and AcGXGNH_2_ peptides. The coupling constants were measured by a peak-fitting procedure to Lorentzian line shape, the fitting results are also shown in the figure. The derived coupling constants can be reproduced within 0.02 Hz if we fit a certain spectrum multiple times independently. In this and our previous studies, we used the Karplus equation by Vuister and Bax^43^ with coefficients: A = 6.51, B = −1.76 and C = 1.60; another parametrization for the Karplus equation with A = 6.98, B = −1.38 and C = 1.72 by Wang and Bax^50^ is believed to be more accurate. Calculated ^3^J_αN_(α) values for ϕ = −60° are coefficient dependent: 4.11 *vs.* 4.16 Hz for two sets of parameters; as a result, the derived α-population differs by ~2%. Given the average difference between ^3^J_αN_ of AcGXPNH_2_ and AcGXGNH_2_ is about 0.41 Hz, plus a maximal uncertainty of 0.2 Hz on ^3^J_αN_(α) due to the uncertainties on the Karplus equation coefficients, we estimate the error of the derived α-population being around 10% for the majority of residues with non-overlapping amide signals, the estimated error could reach to 15–20% for those residues with overlapping peaks.

### The relative population ratio between PII and β for AcGXGNH_2_ and AcGXPNH_2_

In this study, we assume that the population ratio between PII and β is approximately the same for AcGXGNH_2_ and AcGXPNH_2_. It is a known fact that there are secondary neighboring residue effects; we consider the effects from the side chain of residue X itself the primary effects. To our knowledge, Pro as a neighboring residue will make X favoring PII as compared to other neighboring residues. As a result, the population ratio between PII and β cannot be exactly the same for AcGXGNH_2_ and AcGXPNH_2_; it is most likely that the ratio for AcGXPNH_2_ is relatively larger than that for AcGXGNH_2_. Unfortunately, our current understanding on neighboring residue effects remains poor. To investigate the effects, first we define a parameter for the ratio of ratios, RR = GXG_PII/β_/GXP_PII/β_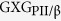  = [x_PII_(GXG)/x_β_(GXG)]/[x_PII_(GXP)/x_β_(GXP)], then we analyze our data systematically with the parameter RR setting from 0.80–1.10 in a step function of 0.05. (Table S2). It is clear that the derived content values shift in the same direction for all residues upon changing the value of RR. Specifically, average contents of PII increase by 1.8%, while average contents of β and α decrease by 0.5% and 1.3%, respectively, upon increasing the parameter RR by 0.05. To our gratification, the correlations and the conclusions hold really well upon changing the value of the parameter RR from 0.80–1.10 (Figs S11–S13).

## Conclusion

We have determined the populations of three major conformers in AcGXGNH_2_ through analyzing ^3^J_αN_ coupling constants of AcGXPNH_2_ and AcGXGNH_2_; the free energy-conformation diagrams are constructed for AcGXGNH_2_ peptides in aqueous solution. Our derived results show that on average residue X in AcGXGNH_2_ adopt PII, β, and α 44.7%, 44.5% and 10.8% of time, respectively. Minor populated α-conformations of different amino acids in AcGXGNH_2_ determine their varying α-helix nucleation capabilities. The contents of α-conformations for different amino acids define an α-helix nucleation propensity scale. There are no correlations observed between the x_α_ values and any α-helix propensity scales^45^,^46^,^47^. Based on our derived β-contents, ΔG values for the corresponding PII to β equilibriums show a reasonable correlation with the β-sheet scale by Kim and Berg^48^, consistent to the observation in AcGGXGGNH_2_ peptides^18^. Derived ΔG values for PII to β transitions show a good correlation to those derived for dipeptides^23^. We have detected significant turn structures in AcGNPNH_2_, AcGTPNH_2_ and AcGDPNH_2_ (pH = 2 and 6)^44^. Results from this study have broad implications on the early-stage events of protein folding. Together with the results on 19 amino acid dipeptides^23^, our results would provide a bench mark for force field developments and for testing predicting calculations of conformational energy maps of flexible model peptides^38^,^39^.

## Methods

Equation (1) was derived by assuming the PII to β population ratio of X in AcGXPNH_2_ and AcGXGNH_2_ being approximately the same. Peptides were synthesized and characterized as described^27^, by using an automated peptide synthesizer with standard Fmoc chemistry. CD spectra were recorded on a J-810 spectrometer with about 100–500 μM peptides in 10 mM phosphate buffer at 25 °C. The concentrations of peptides were determined from a combination of UV absorbance and NMR peak integration^27^. 1D and 2D (TOCSY and NOESY) ^1^H NMR spectra were collected on Bruker AVANCE 400/600 MHz spectrometers at 25 °C. ^3^J_αN_ coupling constants were determined from high resolution 1D spectra. Details are described in Materials and Methods of Supplementary Information.

## Additional Information

**How to cite this article**: Zhou, Y. *et al.* Populations of the Minor α-Conformation in AcGXGNH_2_ and the α-Helical Nucleation Propensities. *Sci. Rep.* **6**, 27197; doi: 10.1038/srep27197 (2016).

## Supplementary Material

Supplementary Information

## Figures and Tables

**Table 1:** Experimentally determined ^3^J_αN_ (298K) of AcGXGNH_2_, AcGXPNH_2_ and amino acid dipeptides and derived α, PII and β-contents for X in AcGXGNH_2_.

Amino acids	^3^J_αN_ (Hz)AcGXGNH_2_	^3^J_αN_ (Hz)AcGXPNH_2_	^3^J_αN_ (Hz)dipeptide**	x_α_ (%) in AcGXGNH_2_	x_PII_ (%) in AcGXGNH_2_	x_β_ (%) in AcGXGN_2_
Tyr	6.83	7.30	7.13	14.7%	44.0%	41.3%
Trp	6.48	6.99	6.91	17.6%	49.0%	33.4%
Thr	7.68	7.60	7.35	4.0%*	36.4%	59.6%
Arg	6.96	7.20	6.85	8.0%	49.7%	42.3%
Gln	6.95	7.24	7.14	9.3%	48.2%	42.5%
Asn	7.63	7.34	7.45	2.5%*	39.8%	57.7%
Met	7.03	7.26	7.02	7.2%	48.8%	44.0%
Leu	6.81	7.27	6.88	14.5%	44.7%	40.8%
Lys	6.92	7.19	6.83	8.6%	49.8%	41.6%
Ile	7.24	7.91	7.33	17.6%	29.6%	52.8%
Cys	7.25	7.32	7.31	2.2%	49.8%	48.0%
His	7.29	7.95	7.83	17.0%	29.0%	54.0%
Ala	5.86	6.20	6.06	16.3%	66.9%	16.8%
Phe	7.02	7.36	7.18	10.4%	44.8%	44.8%
Ser	6.86	7.19	7.02	10.7%	48.6%	40.7%
Val	7.20	7.91	7.30	18.7%	29.1%	52.2%
Glu (pH = 2)	6.96	7.54	7.02 (pH = 2.9)	16.9%	37.8%	45.3%
Glu (pH = 6)	6.38	6.71	6.63 (pH = 4.9)	12.7%	58.3%	29.0%
Asp (pH = 2)	7.63	7.54	7.51 (pH = 2.9)	2.0%*	40.2%	57.8%
Asp (pH = 6)	7.11	6.51	6.93 (pH = 4.9)	5.0%*	49.8%	45.2%

^*^^*^^3^J_αN_ values of amino acid dipeptides are taken from ref. 19. ^*^Corresponding α content values are taken from ref. 23.

**Table 2:** Derived turn, PII and β-contents of X in AcGXPNH_2_ for Thr, Asn and Asp (pH = 2 and 6).

AcGXPNH_2_	x_turn_ (%)	x_PII_ (%)	x_β_ (%)
Thr	6.1%	35.6%	58.3%
Asn	10.4%	36.6%	53.0%
Asp (pH = 2)	4.7%	39.1%	56.2%
Asp (pH = 6)	24.0%	39.8%	36.2%
