# Supplementary material for: Populations of the Minor α-Conformation in AcGXGNH2 and the α-Helical Nucleation Propensities
Source: Sci Rep. 2016 Jun 3;6:27197. doi: 10.1038/srep27197 (PMC4891685; doi:10.1038/srep27197)
Supplement: Supplementary Information [file srep27197-s1.doc]

**Supplementary Information**

**Populations of the Minor α-Conformation in AcGXGNH_2_ and the α-Helical Nucleation Propensities**

Yanjun Zhou, Liu He, Wenwen Zhang, Jingjing Hu and Zhengshuang Shi*

School of Chemistry and Chemical Engineering, Huazhong University of Science and Technology, 1037 Luoyu Road, Wuhan 430074, P.R. China

*Corresponding author: [zs_shi@hust.edu.cn](mailto:zs_shi@hust.edu.cn)

| 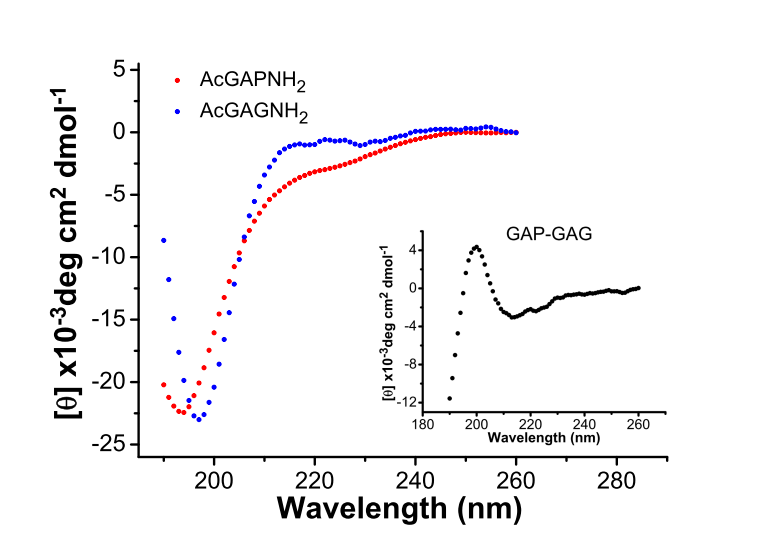 | 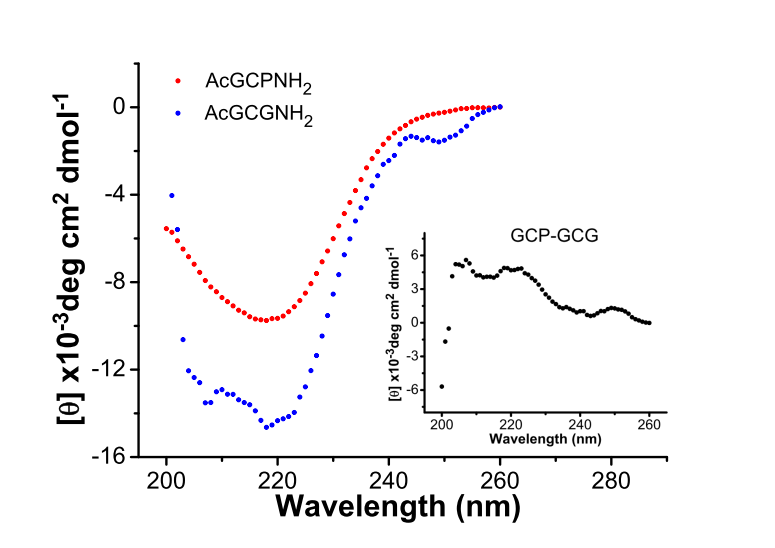 |
| --- | --- |
| 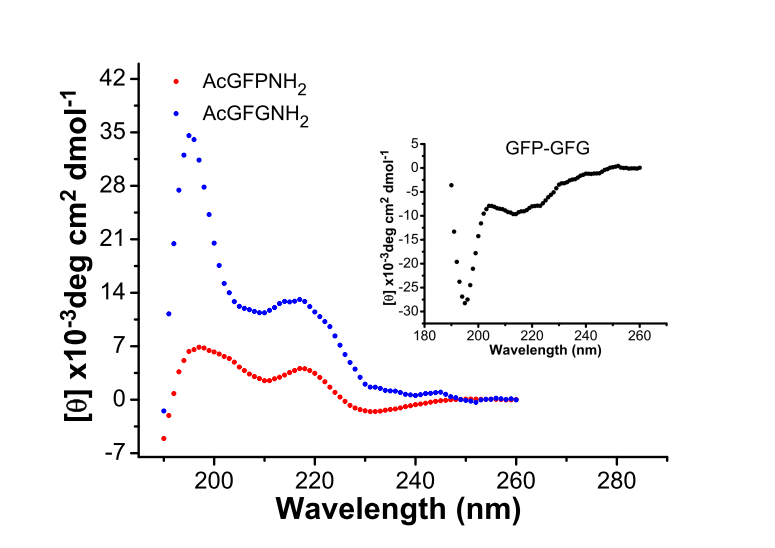 | 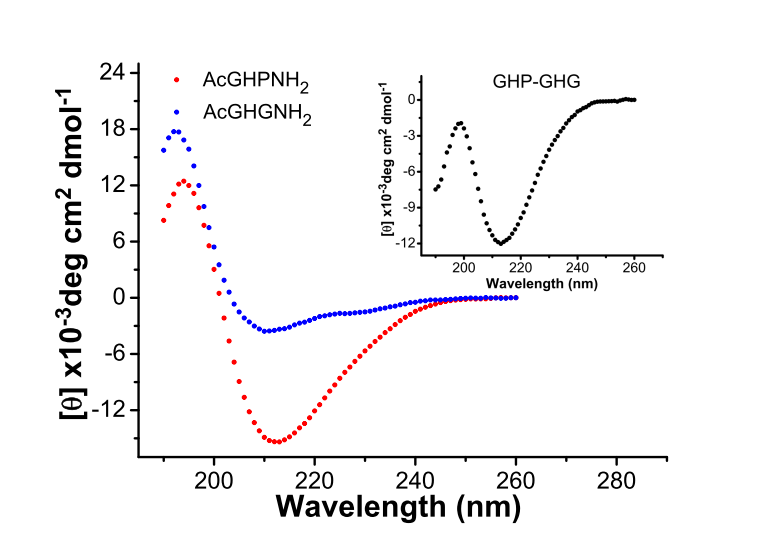 |
| 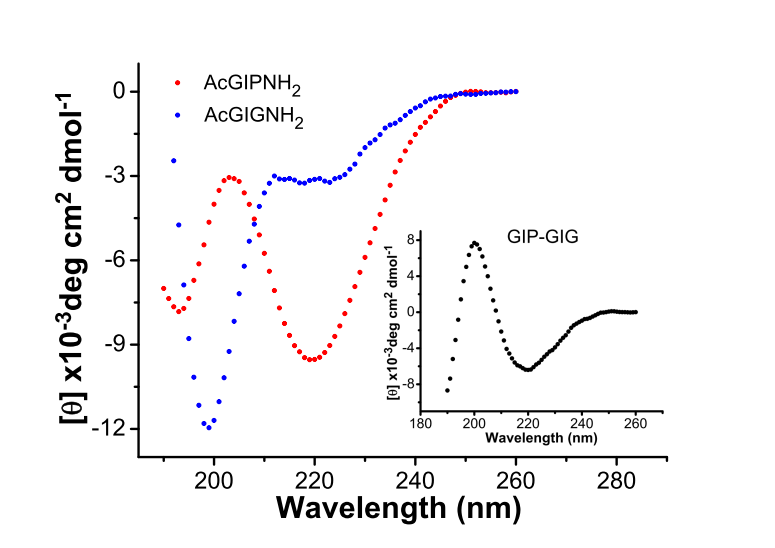 | 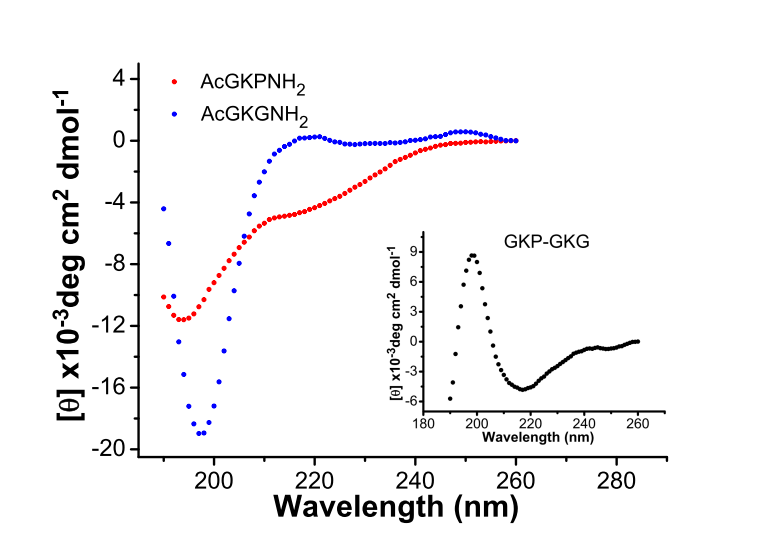 |
| 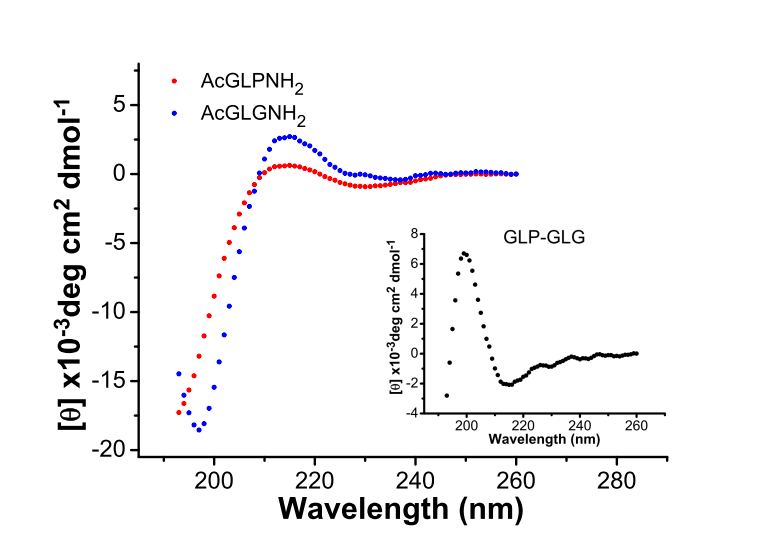 | 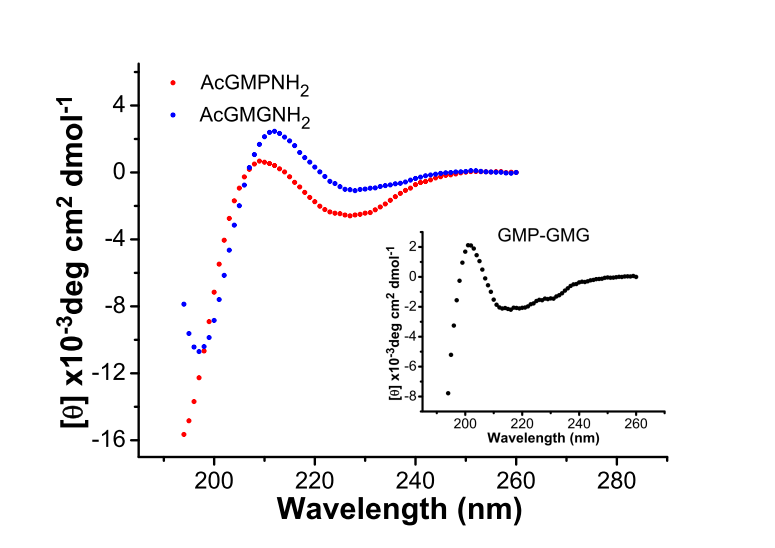 |
| 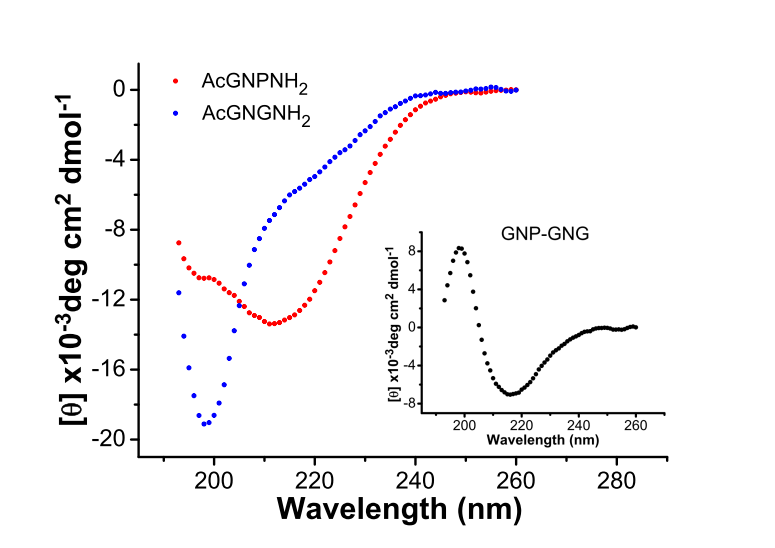 | 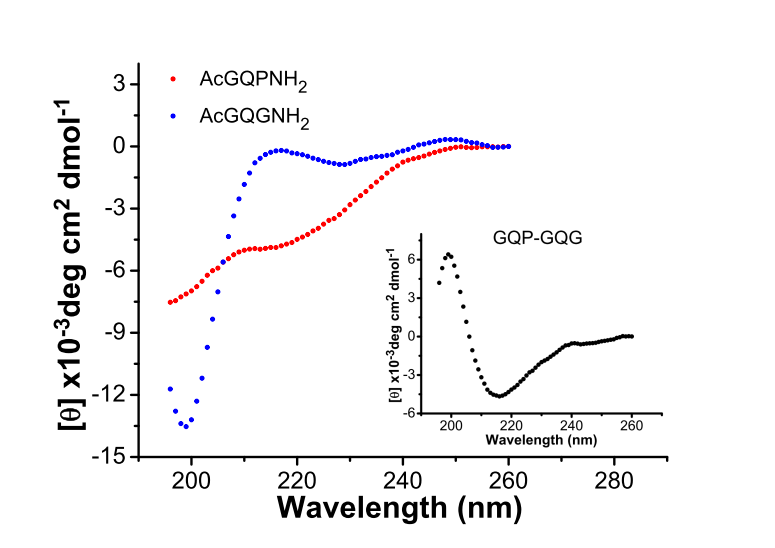 |
| 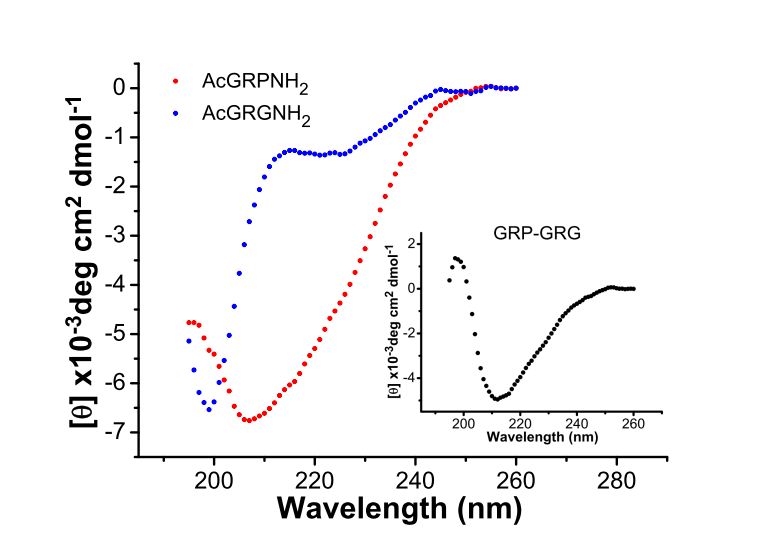 | 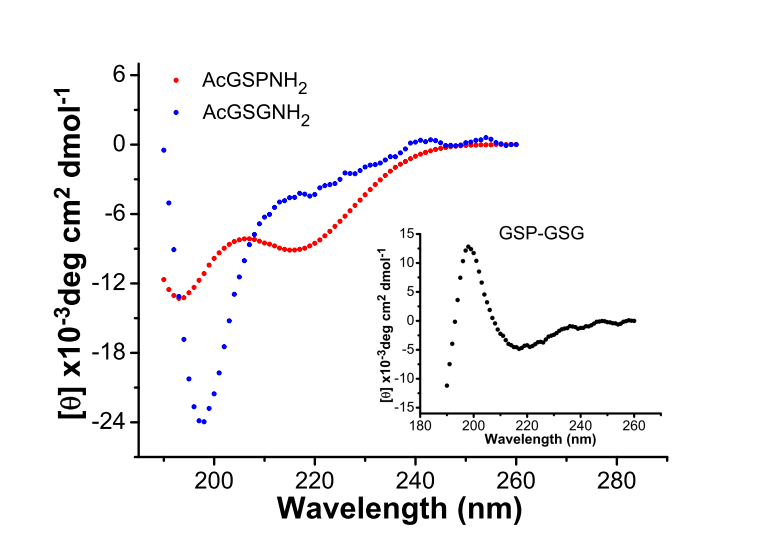 |
| 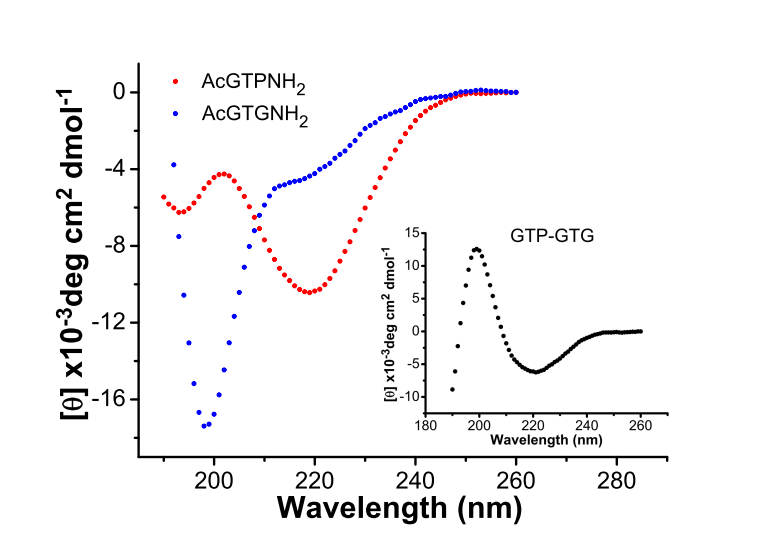 | 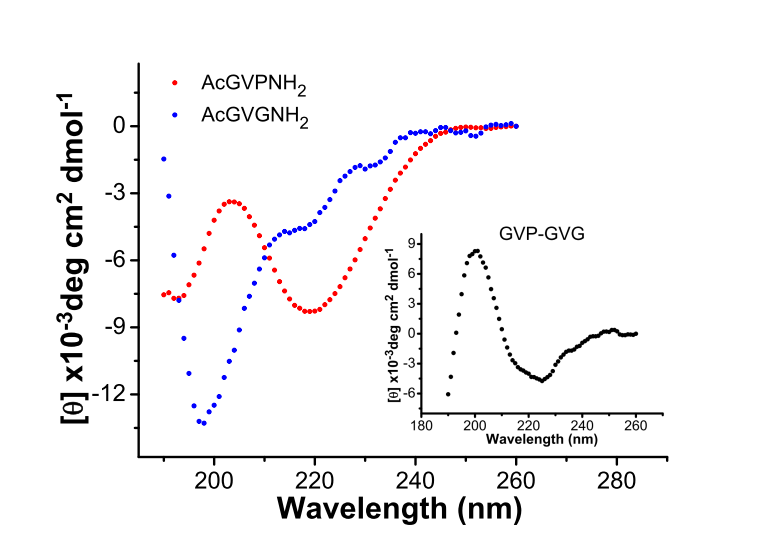 |
| 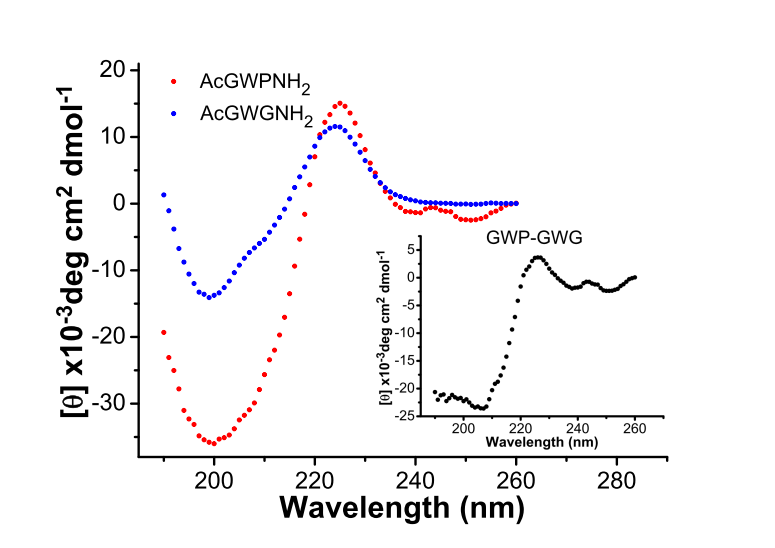 | 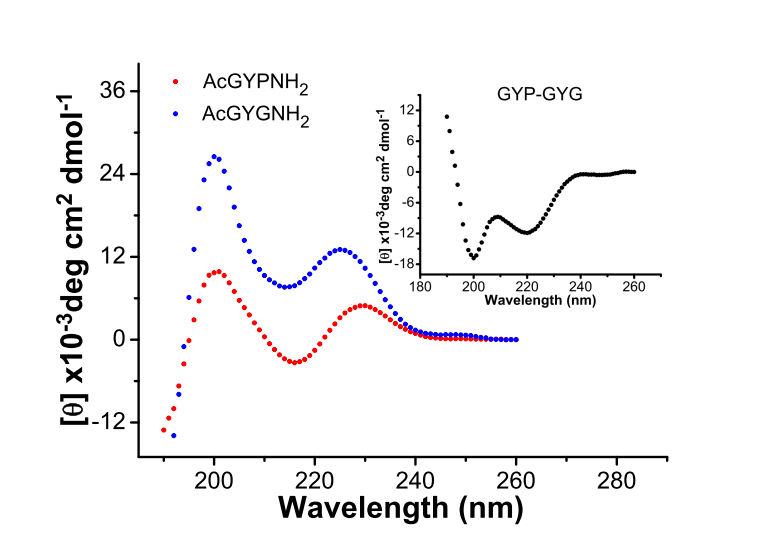 |
| 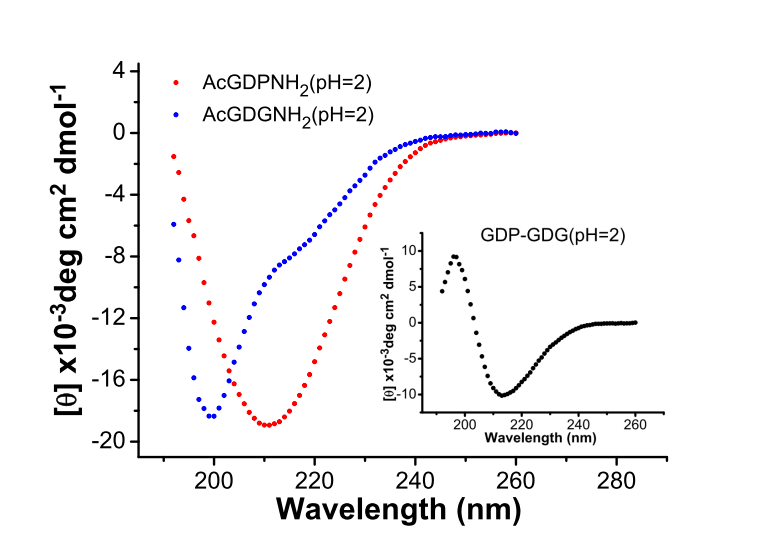 | 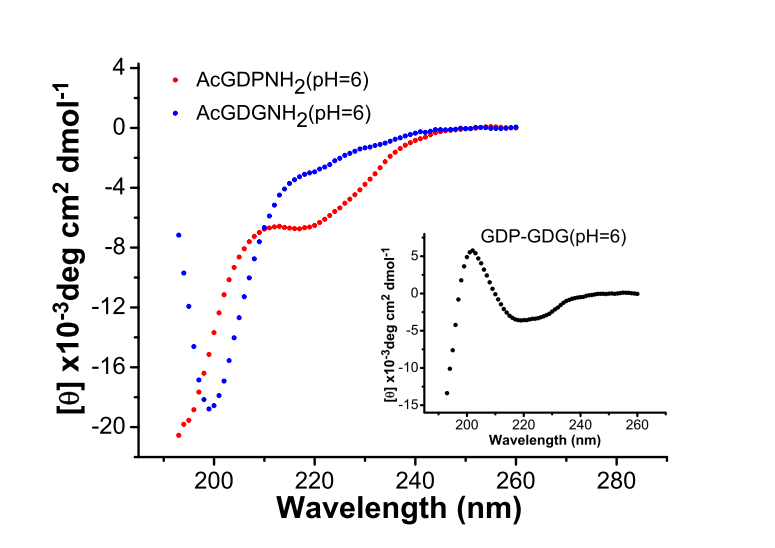 |
| 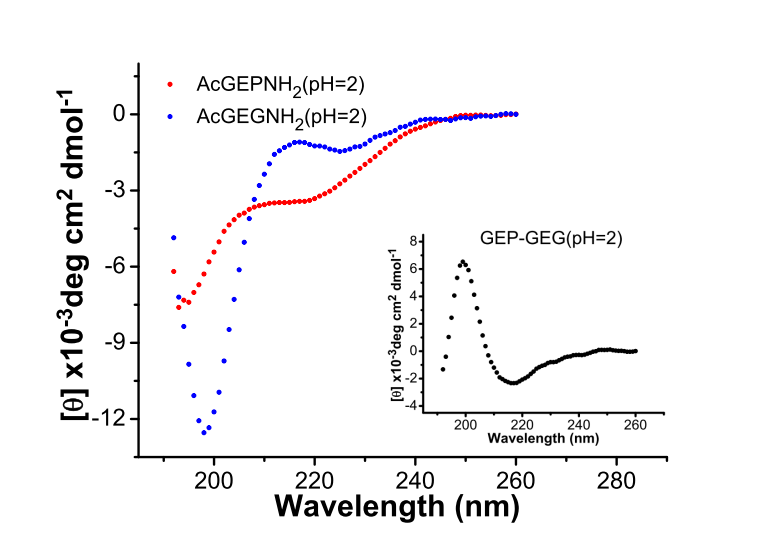 | 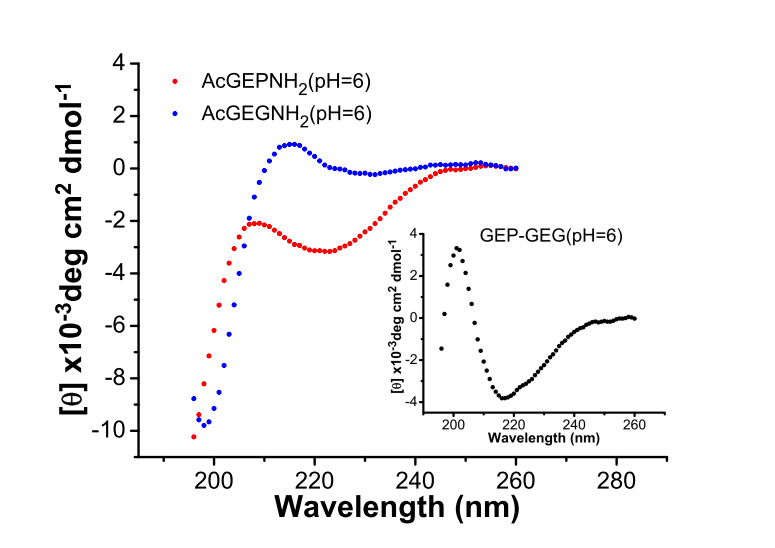 |

**Figure S1** CD spectra of AcGXGNH_2_ (blue) and AcGXPNH_2_ (red) peptides at 25°C. Differential CD spectra between AcGXPNH_2_ and AcGXGNH_2_ are shown in insets.

| 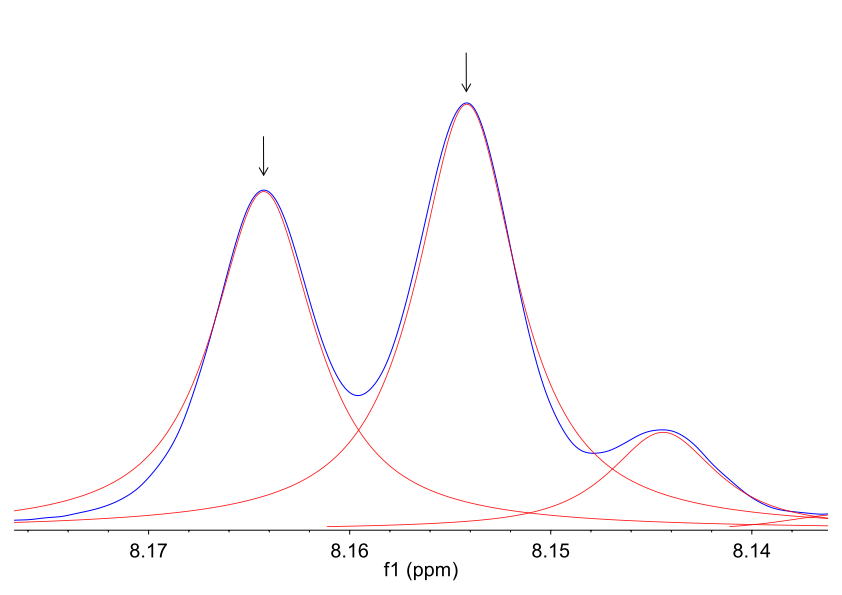 | 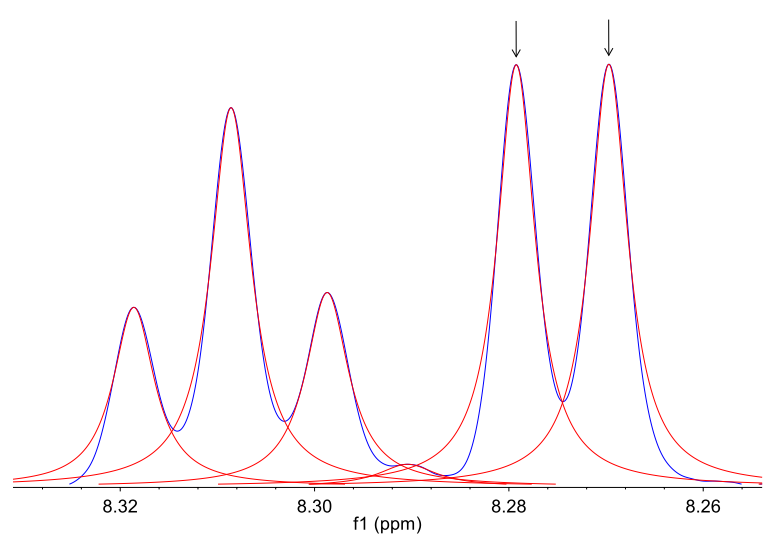 |
| --- | --- |
| GAP | GAG |
| 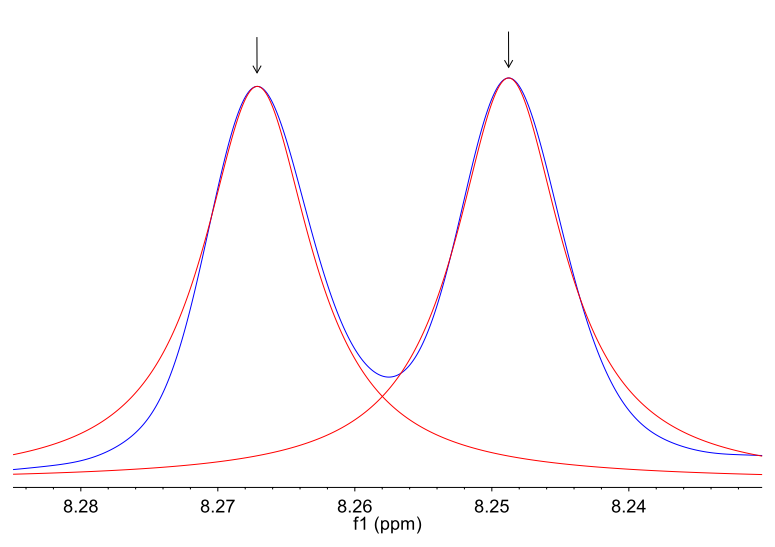 | 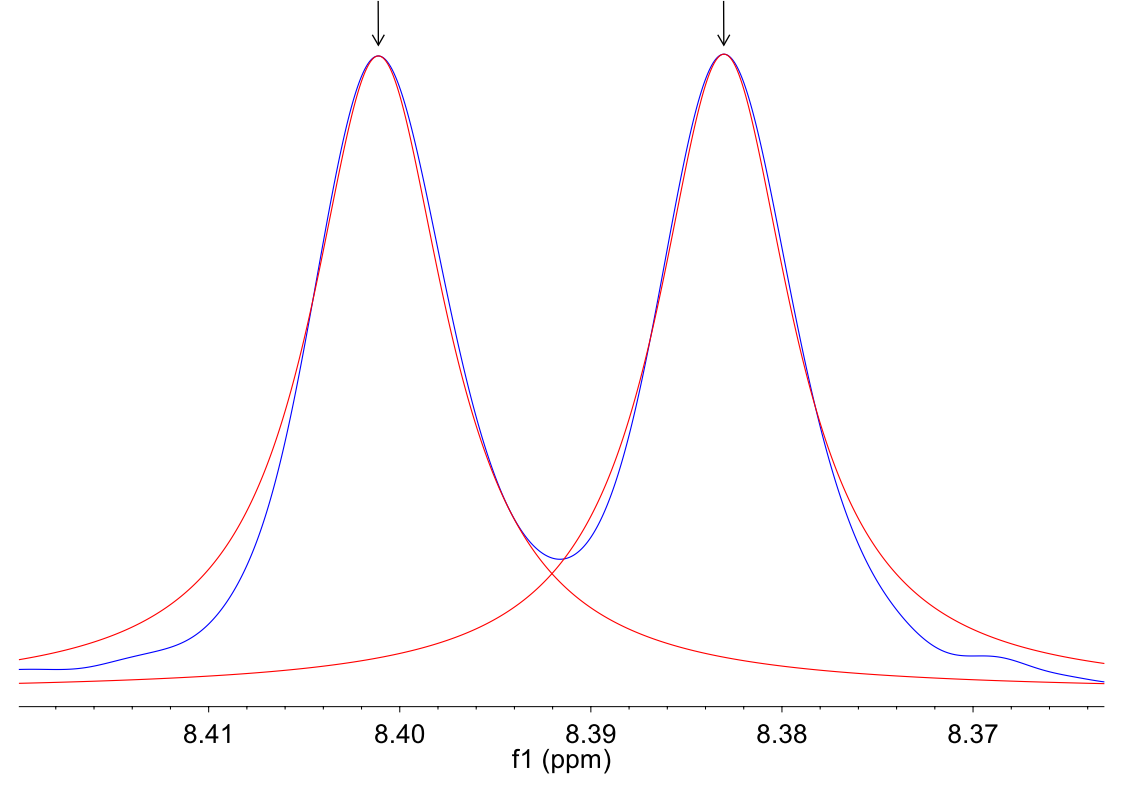 |
| GCP | GCG |
| 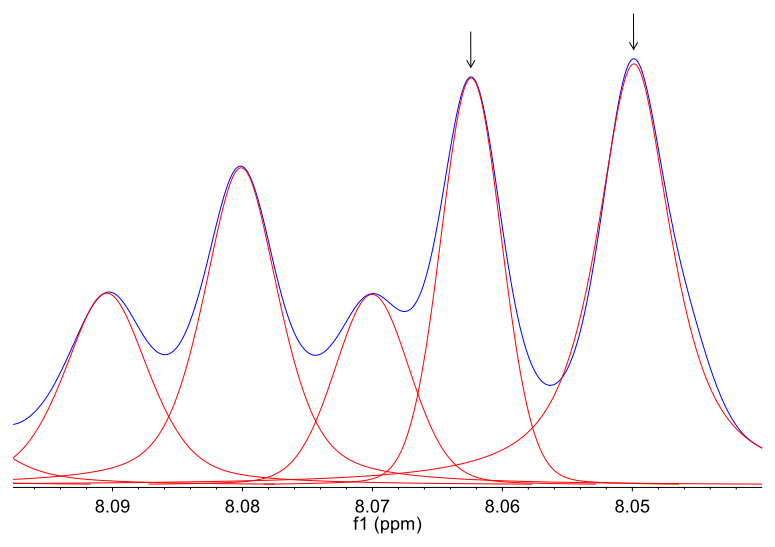 | 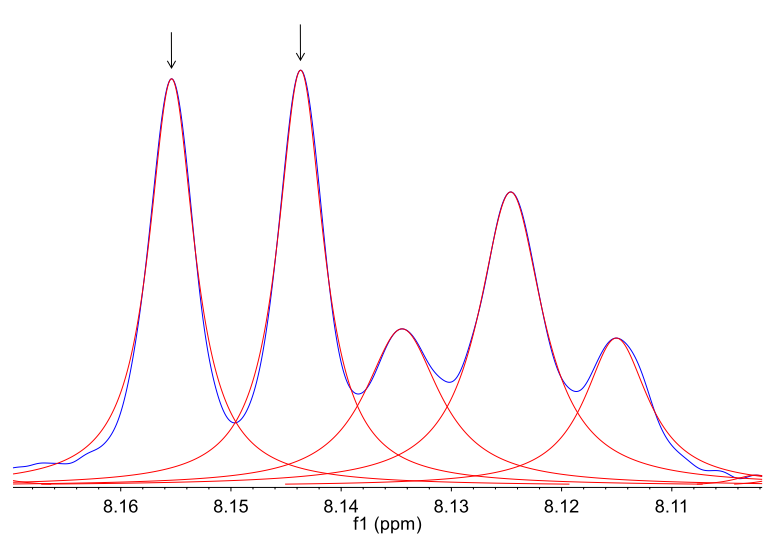 |
| GFP | GFG |
| 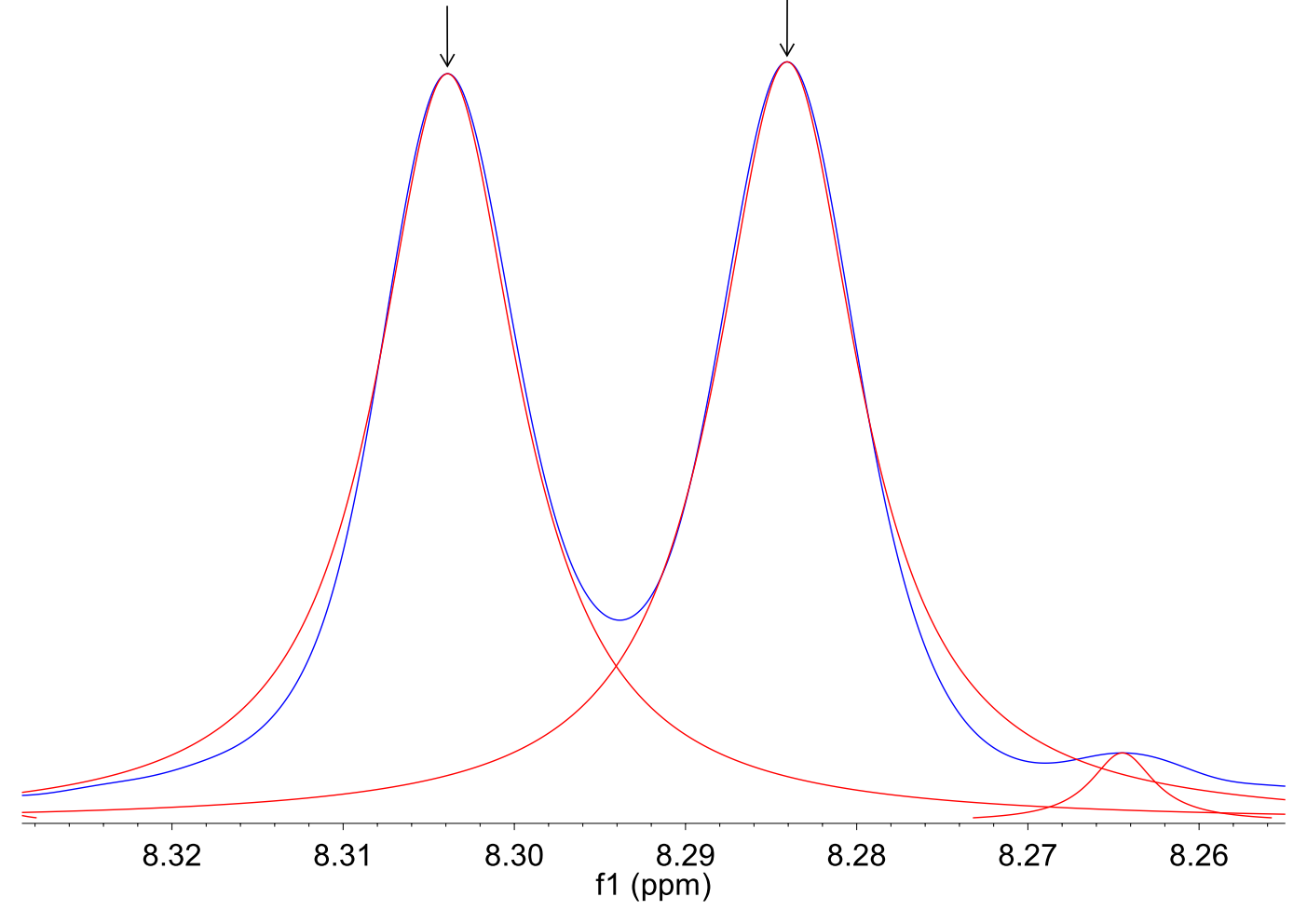 | 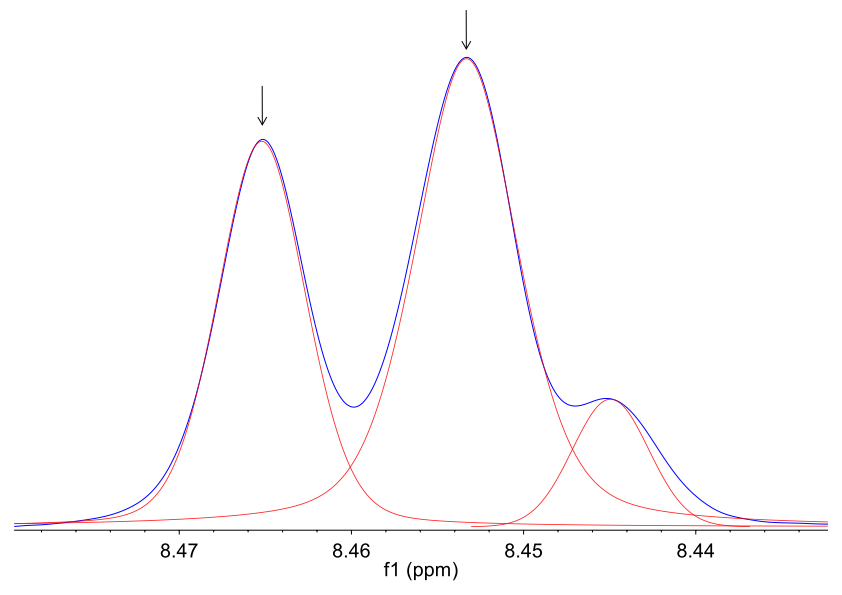 |
| GHP | GHG |
| 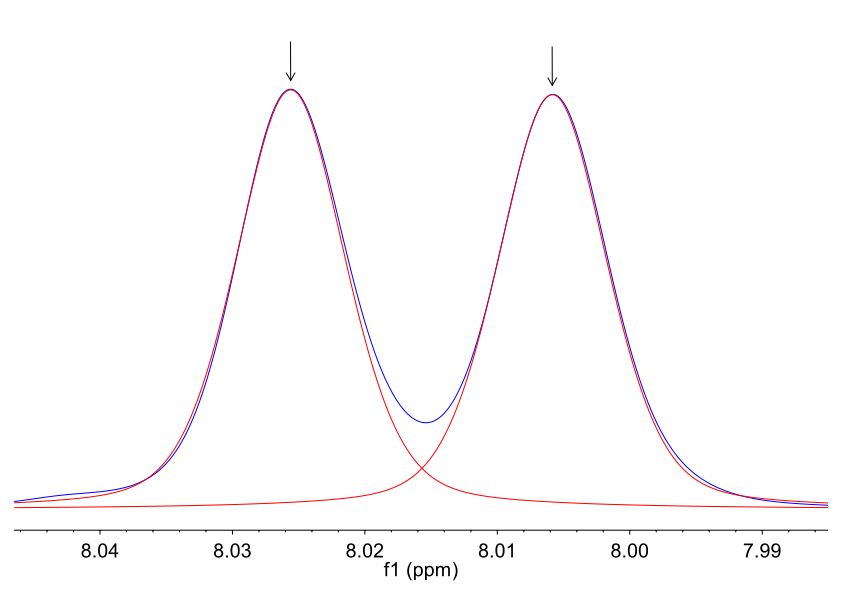 | 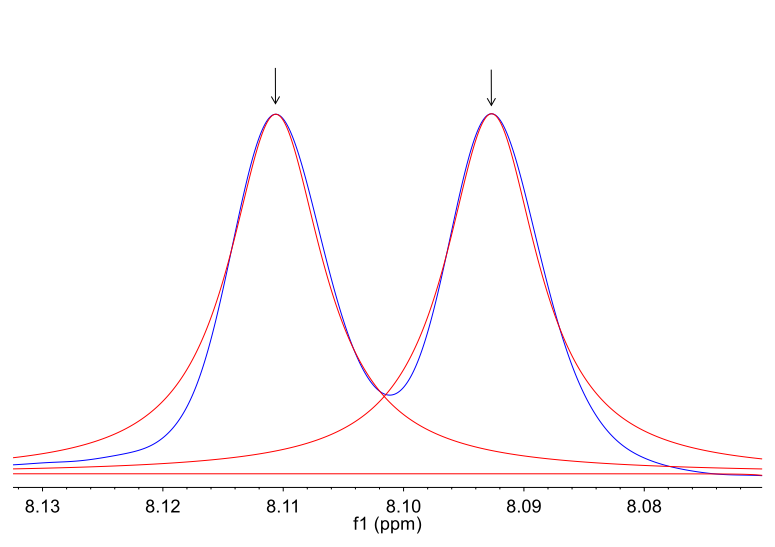 |
| GIP | GIG |
| 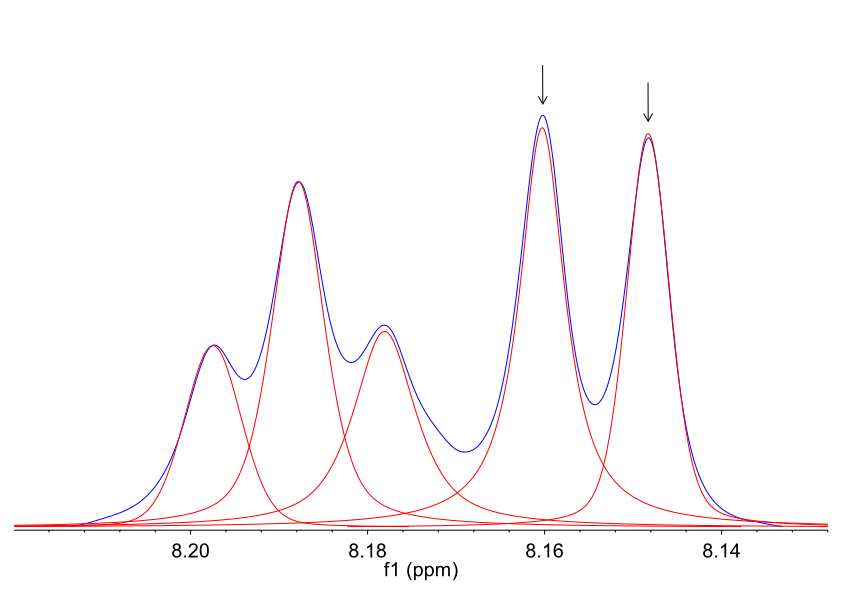 | 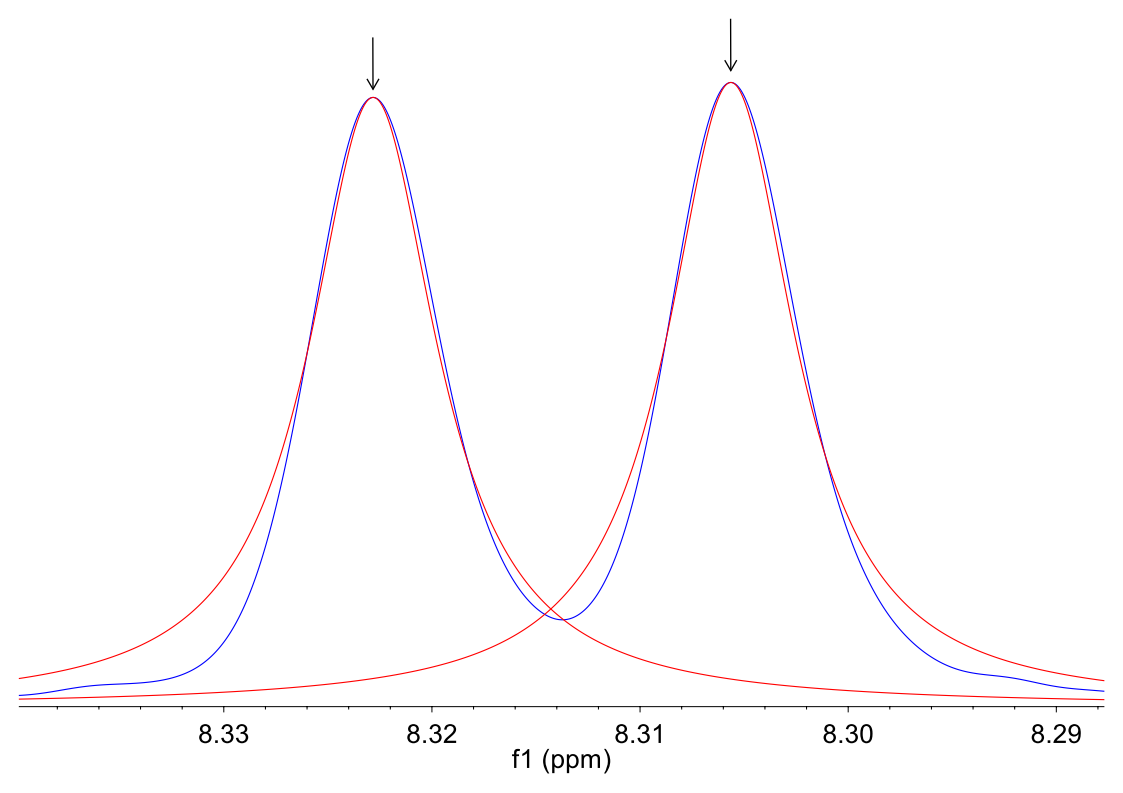 |
| GKP | GKG |
| 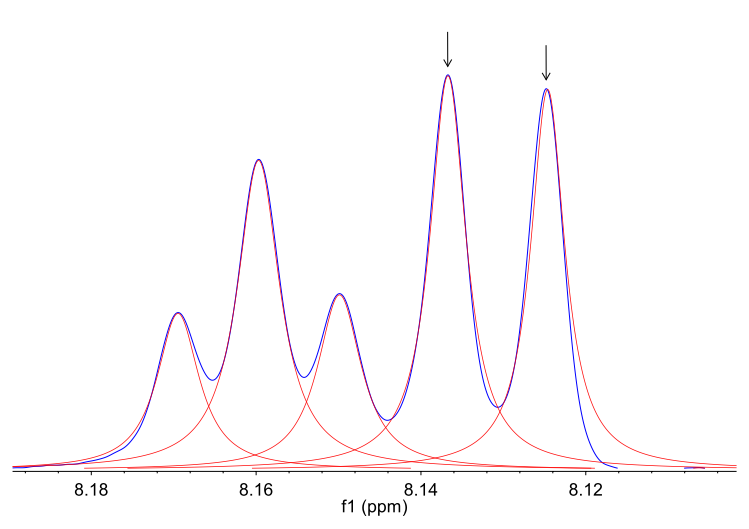 | 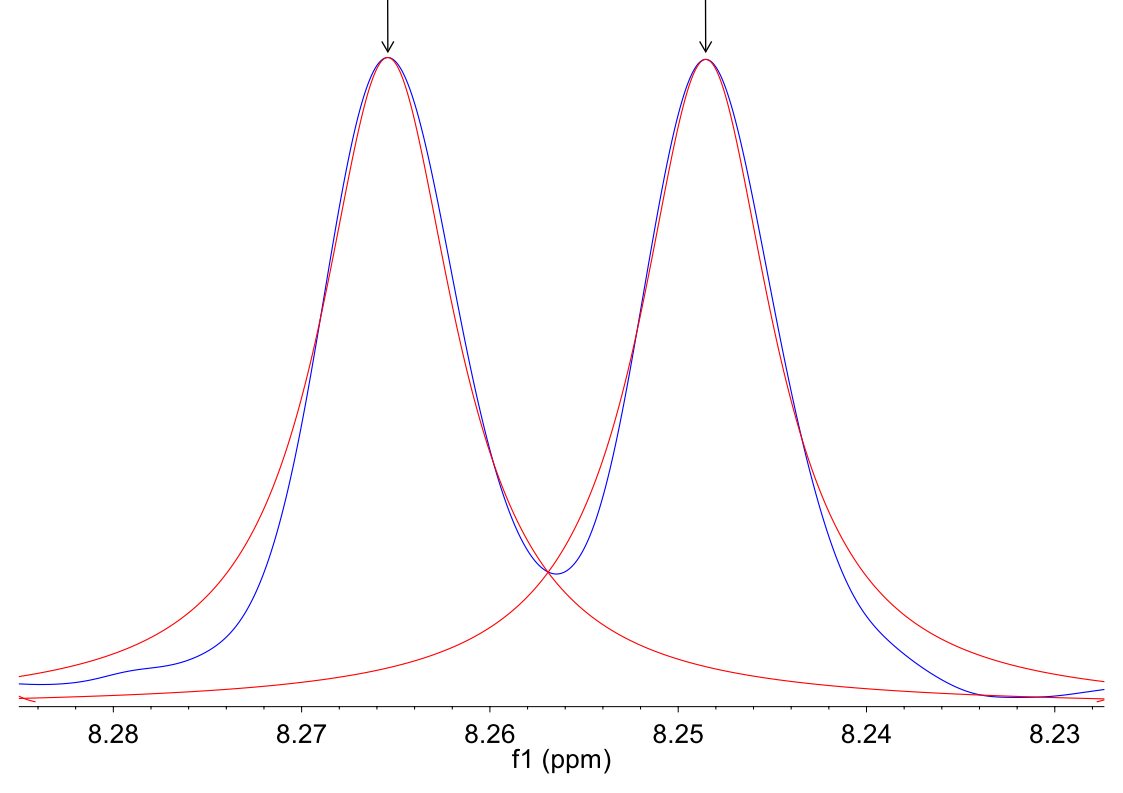 |
| GLP | GLG |
| 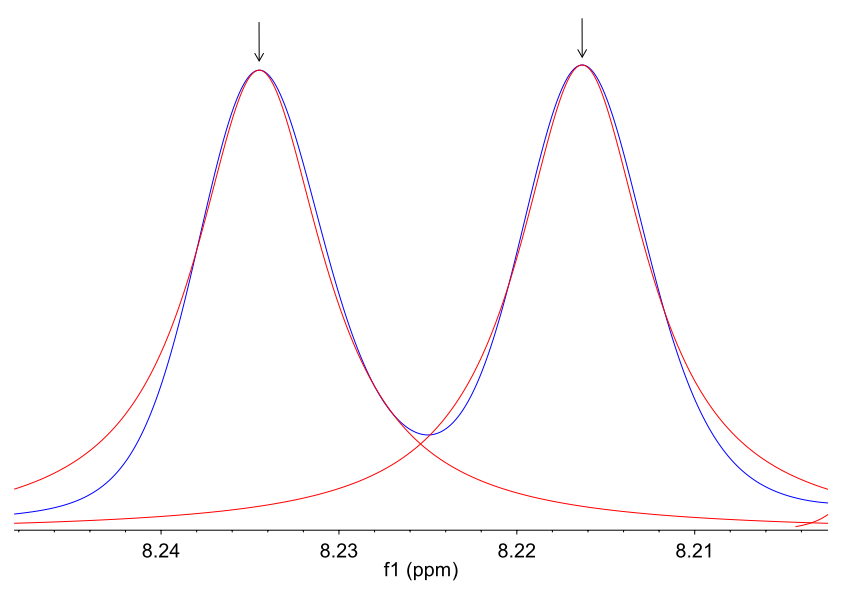 | 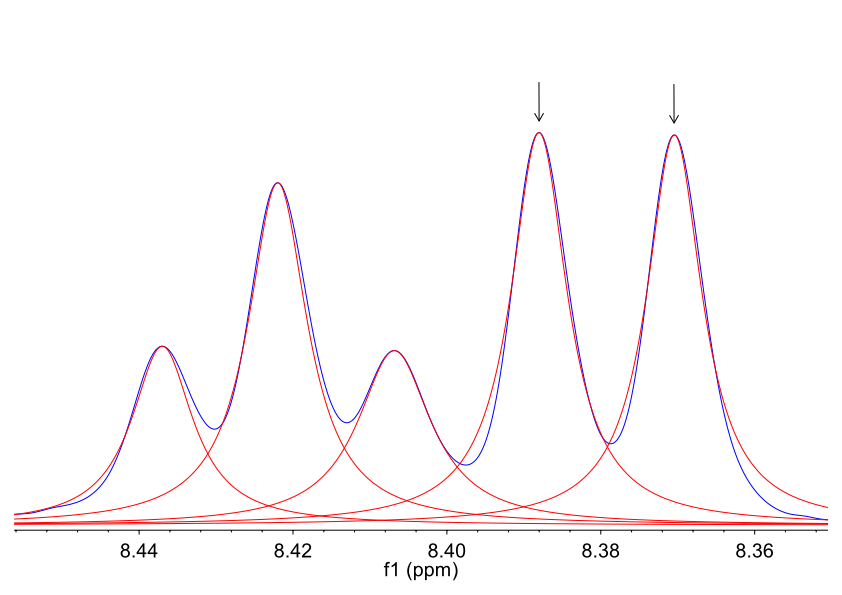 |
| GMP | GMG |
| 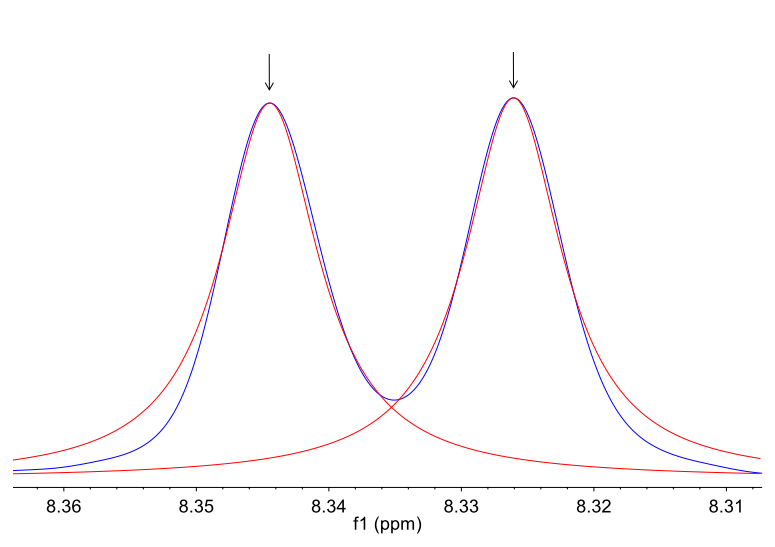 | 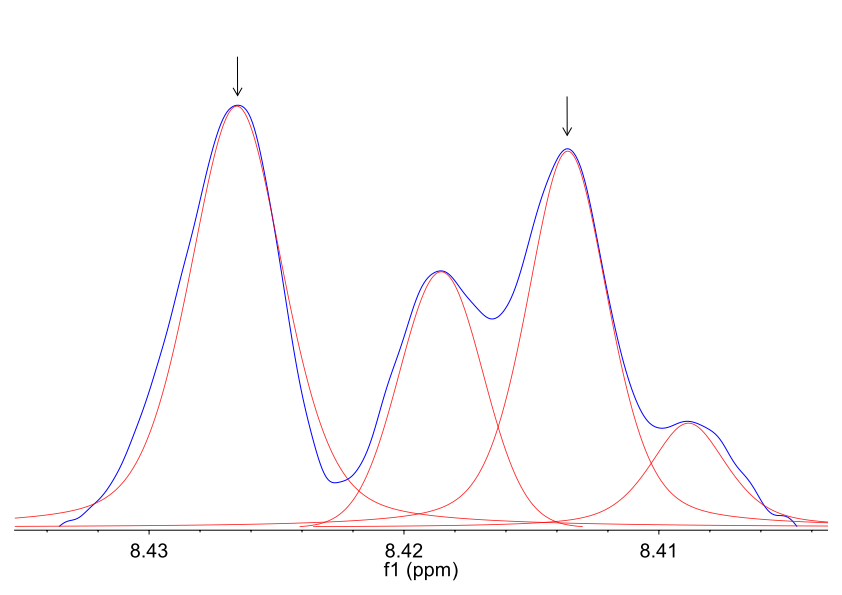 |
| GNP | GNG |
| 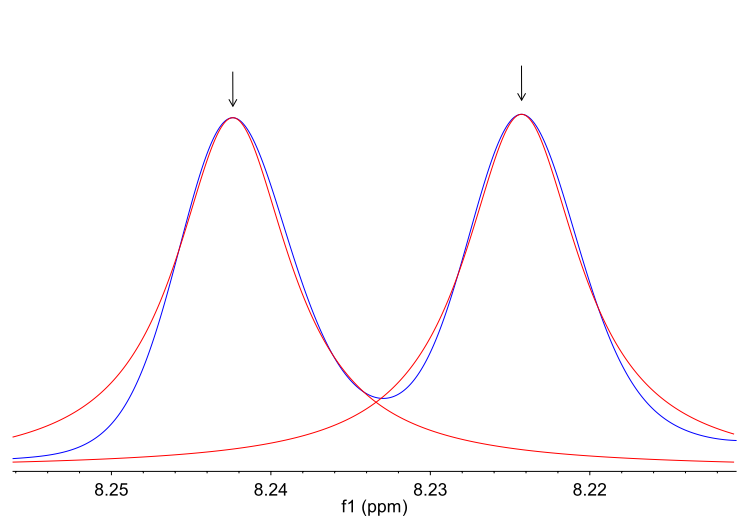 | 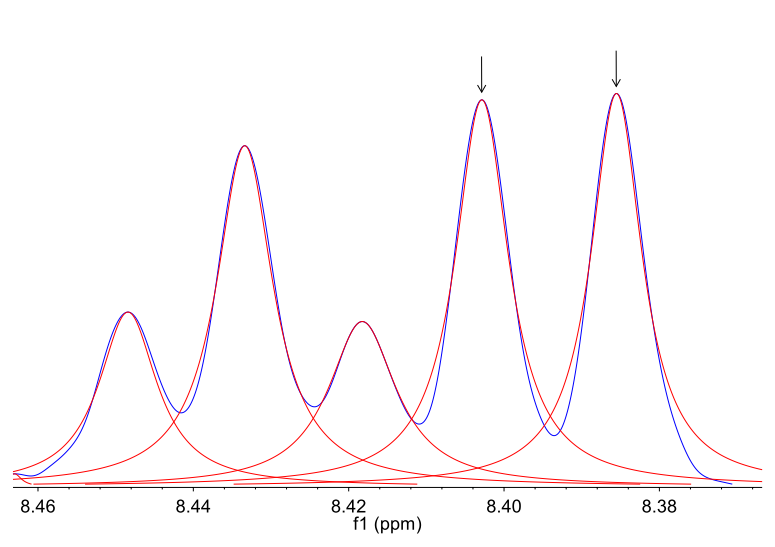 |
| GQP | GQG |
| 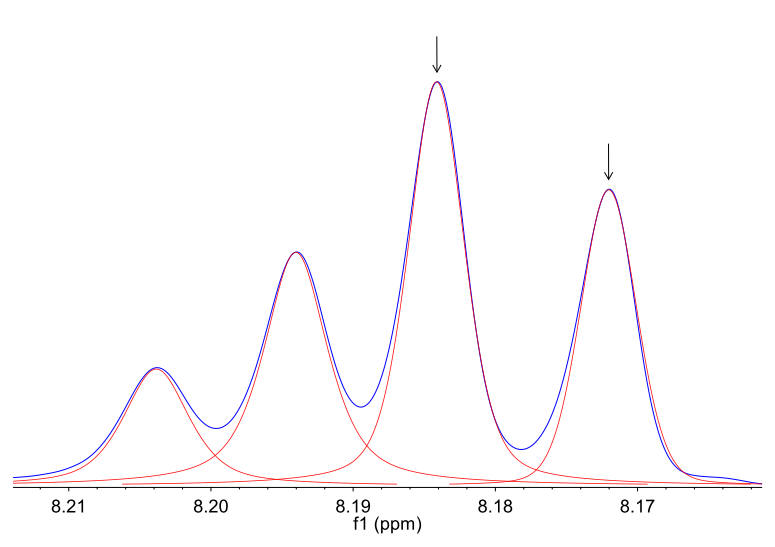 | 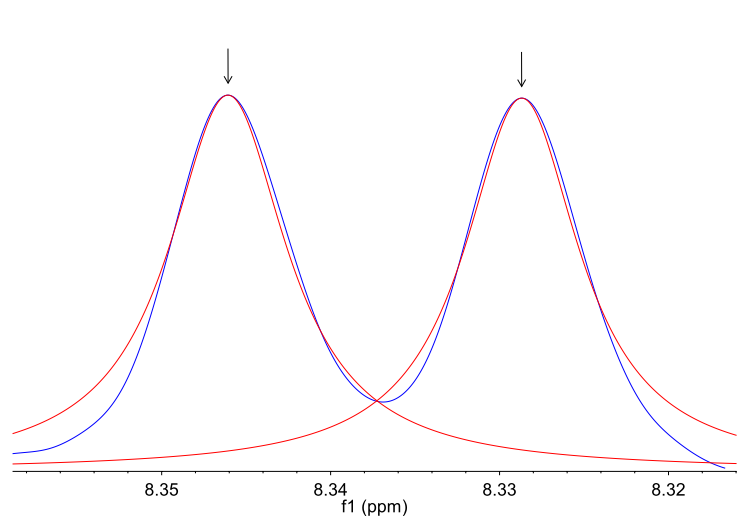 |
| GRP | GRG |
| 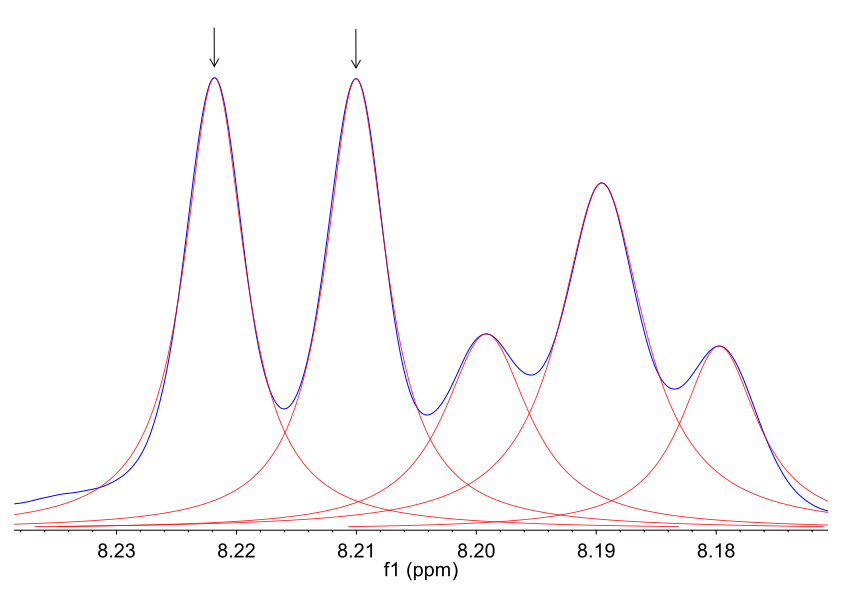 | 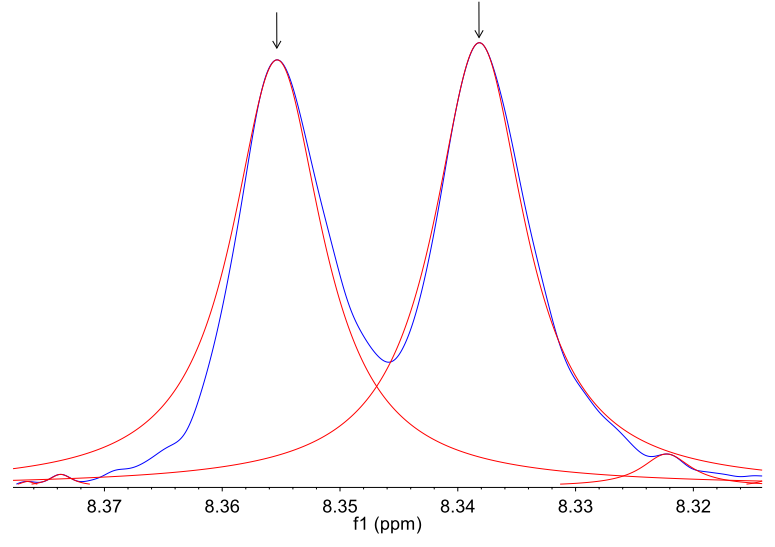 |
| GSP | GSG |
| 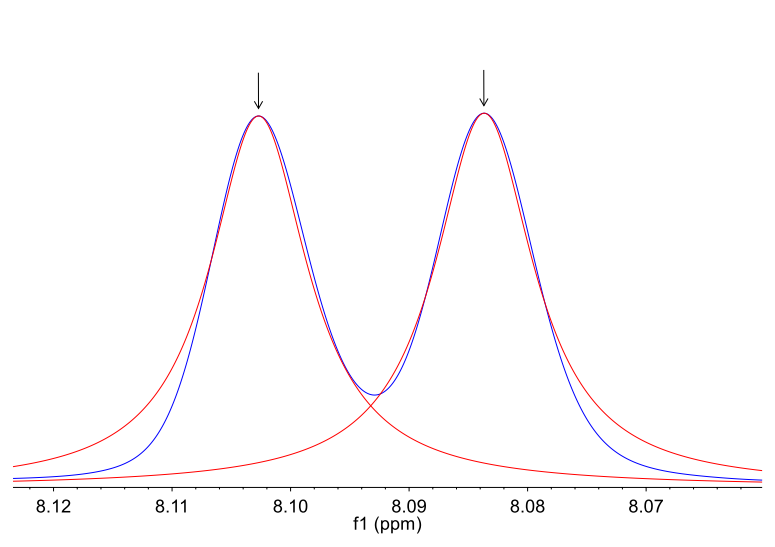 | 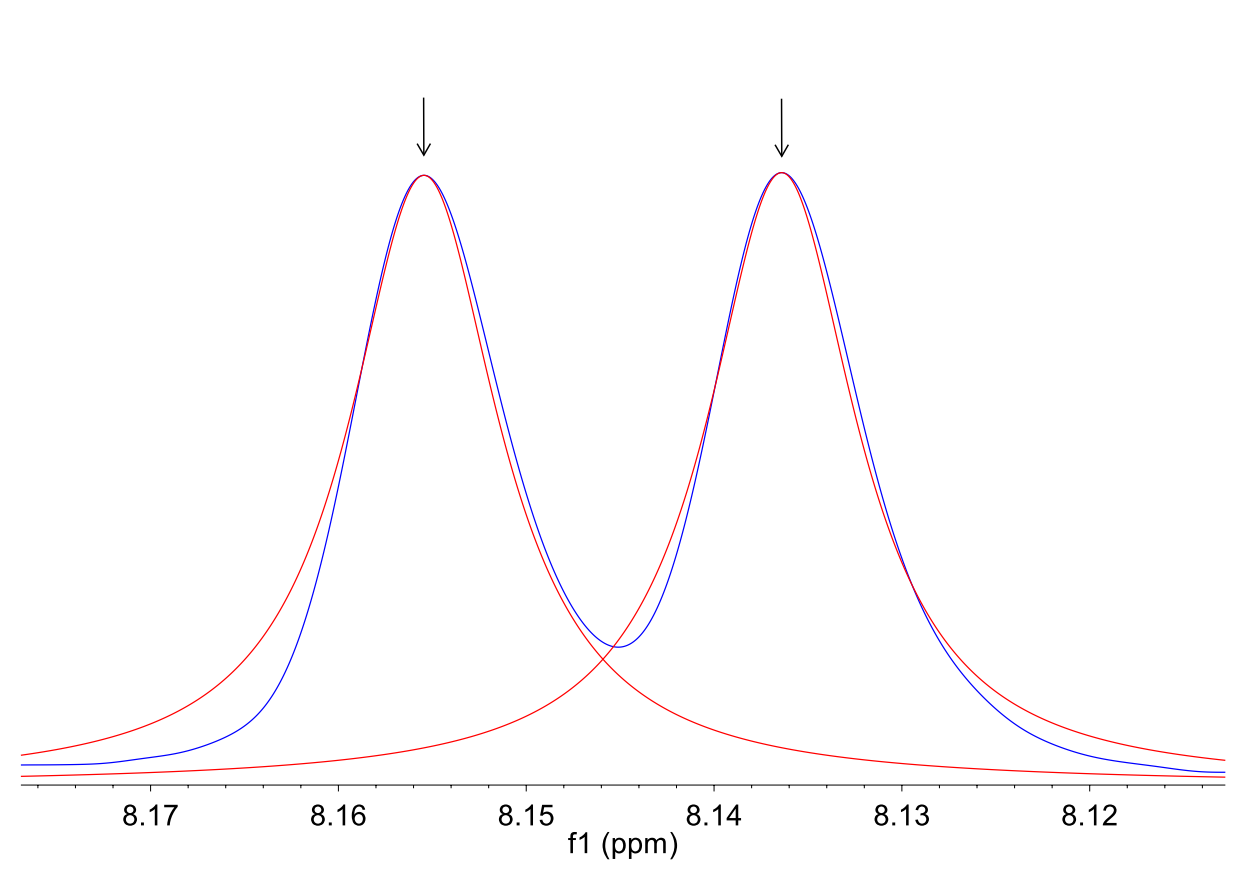 |
| GTP | GTG |
| 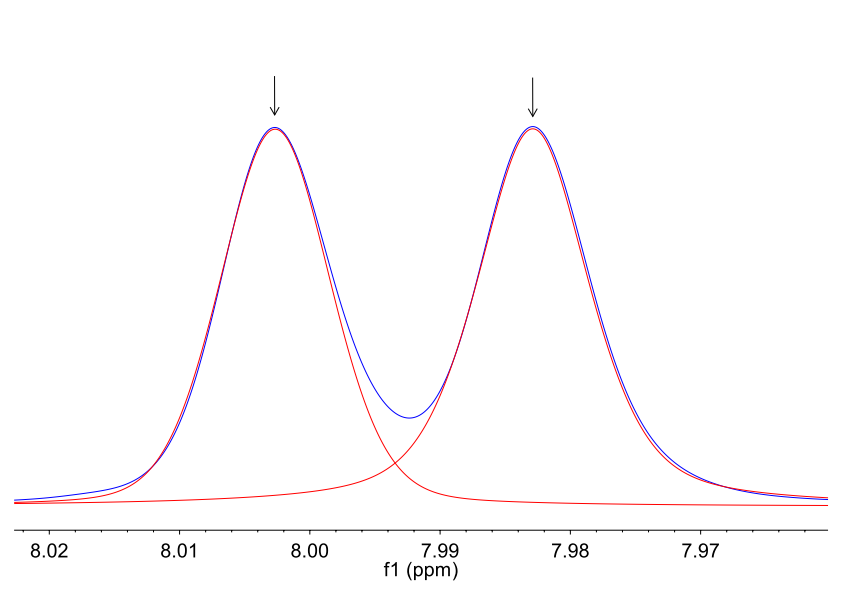 | 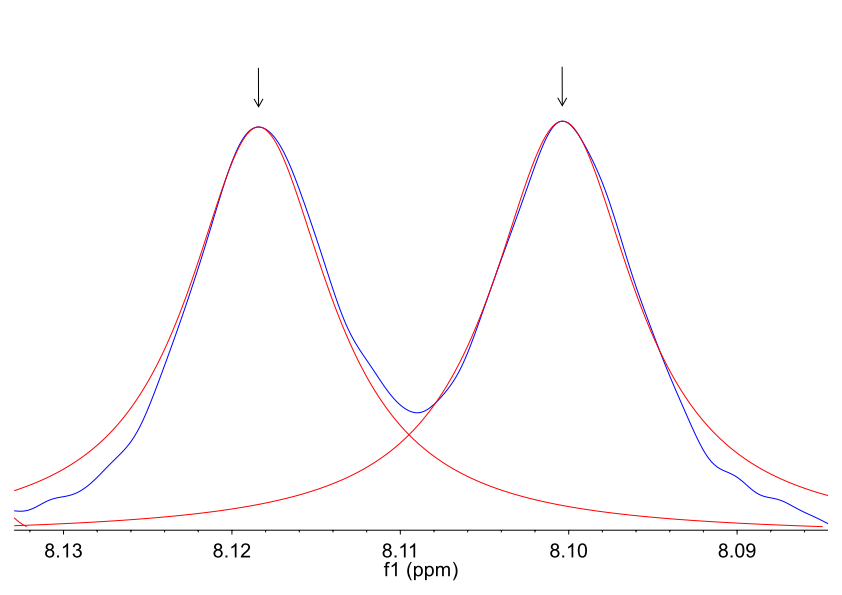 |
| GVP | GVG |
| 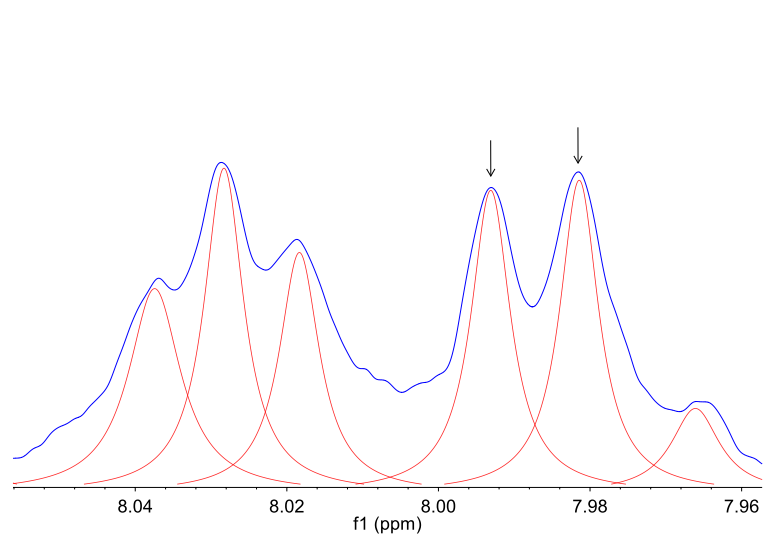 | 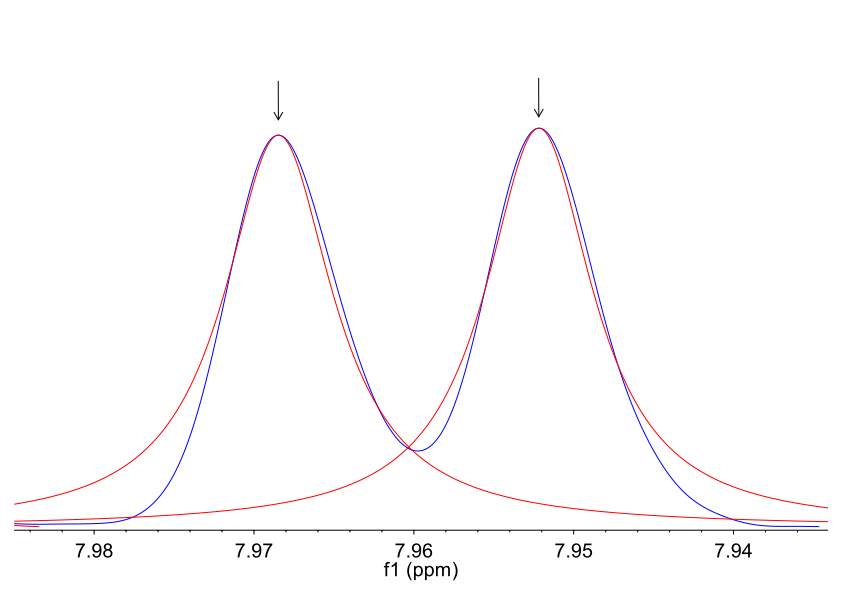 |
| GWP | GWG |
| 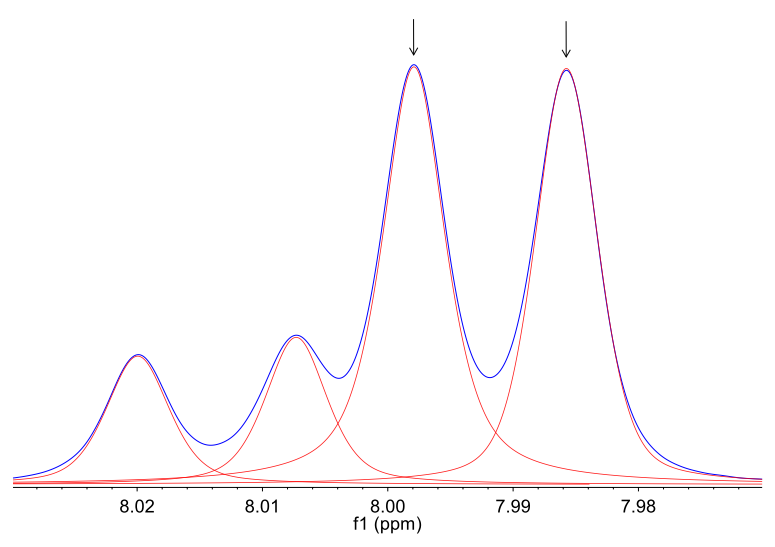 | 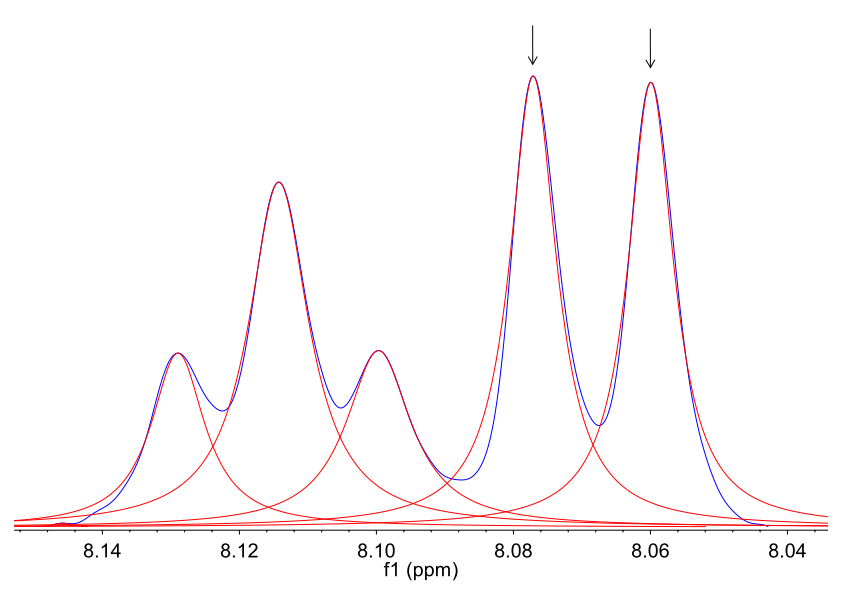 |
| GYP | GYG |
| 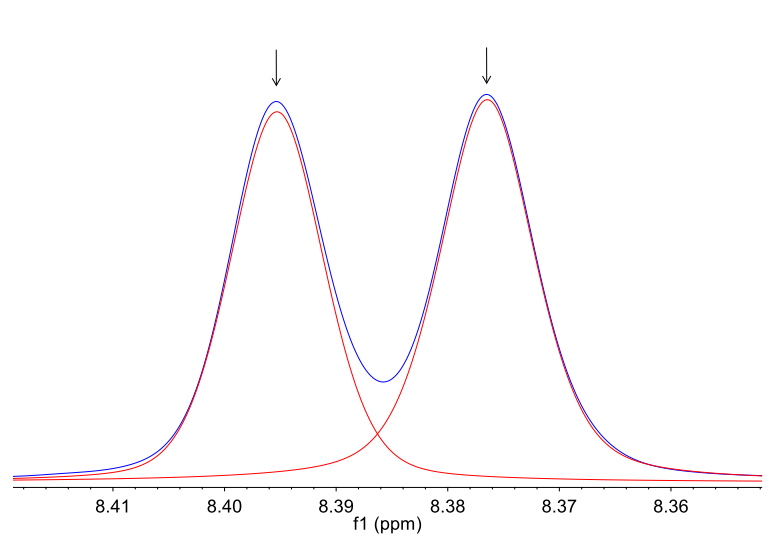 | 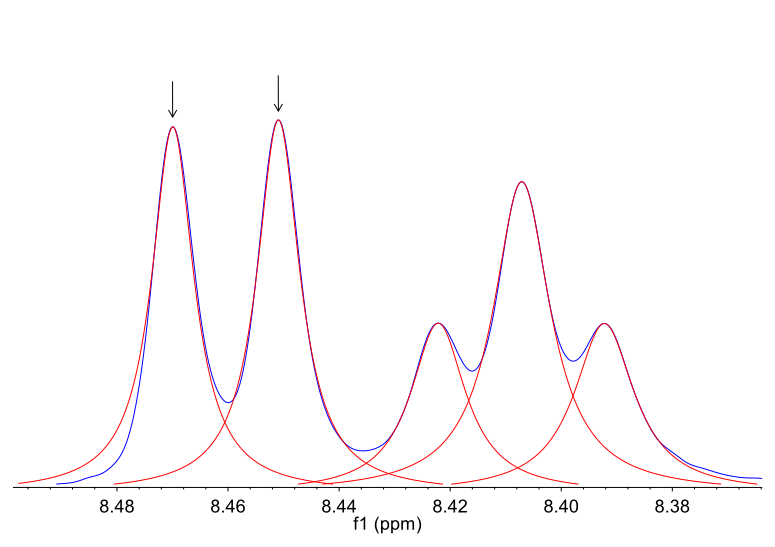 |
| GDP(pH=2) | GDG(pH=2) |
| 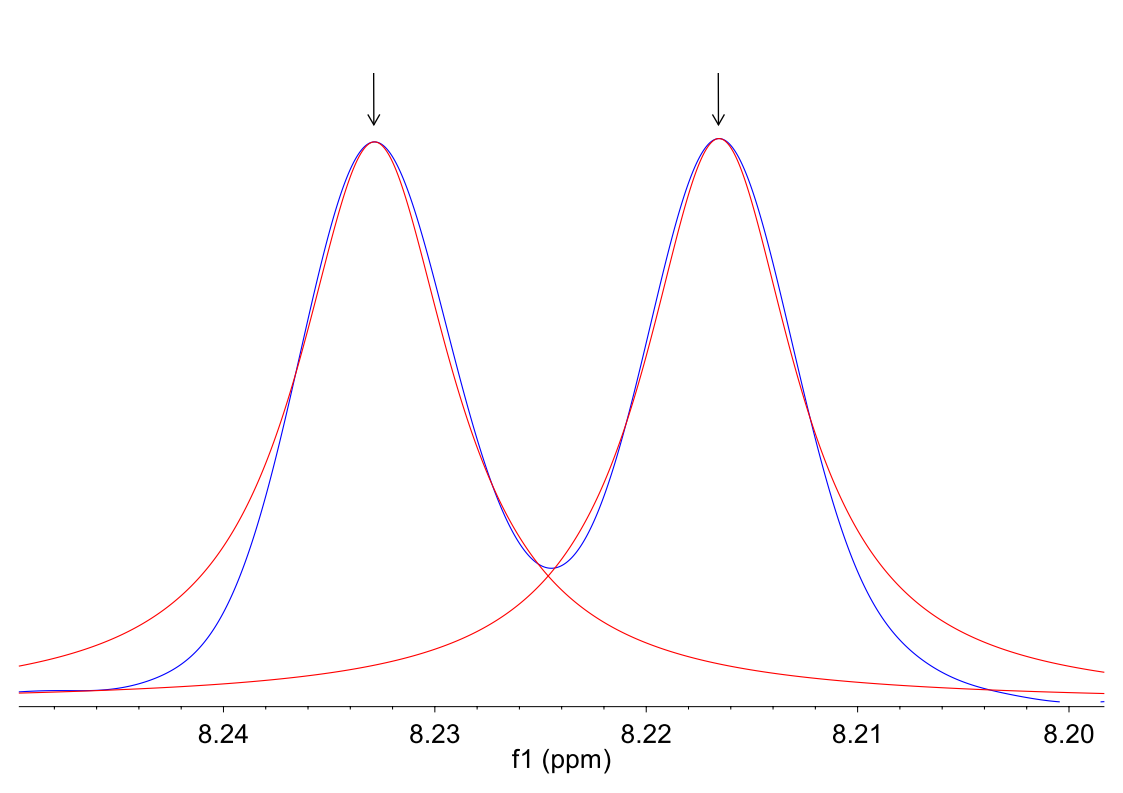 | 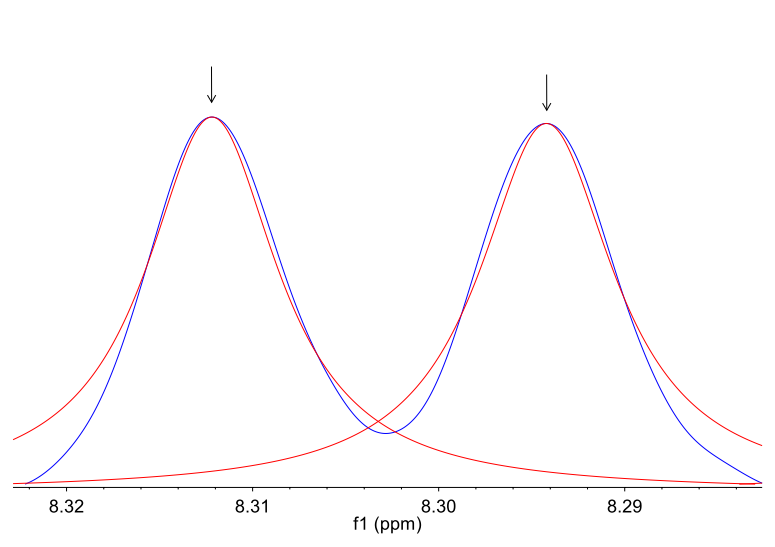 |
| GDP(pH=6) | GDG(pH=6) |
| 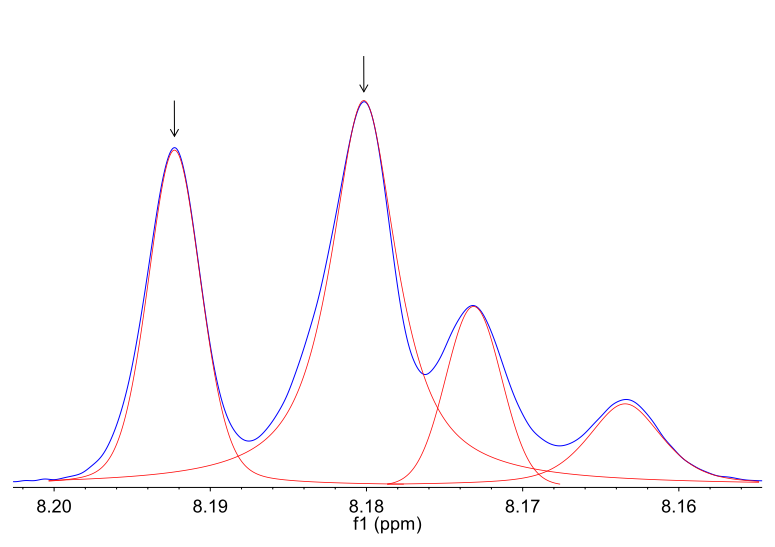 | 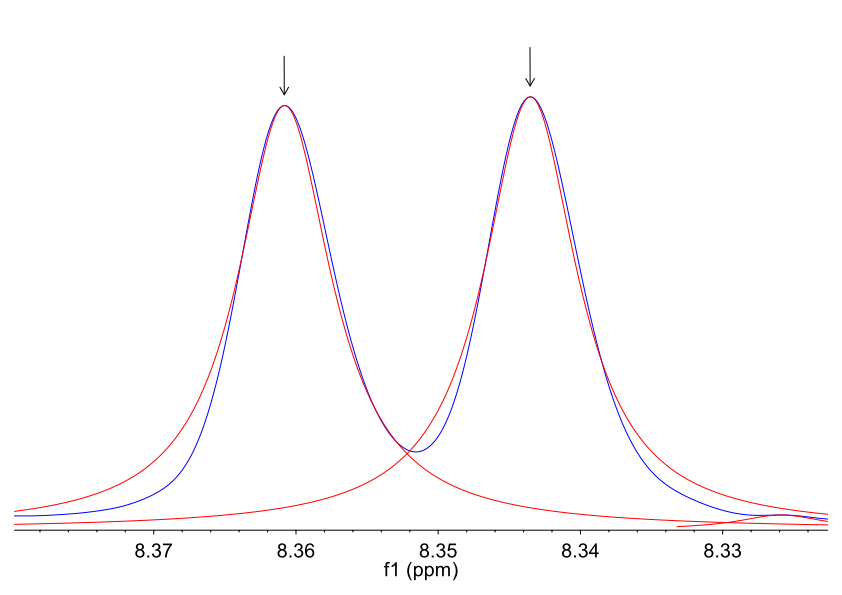 |
| GEP(pH=2) | GEG(pH=2) |
| 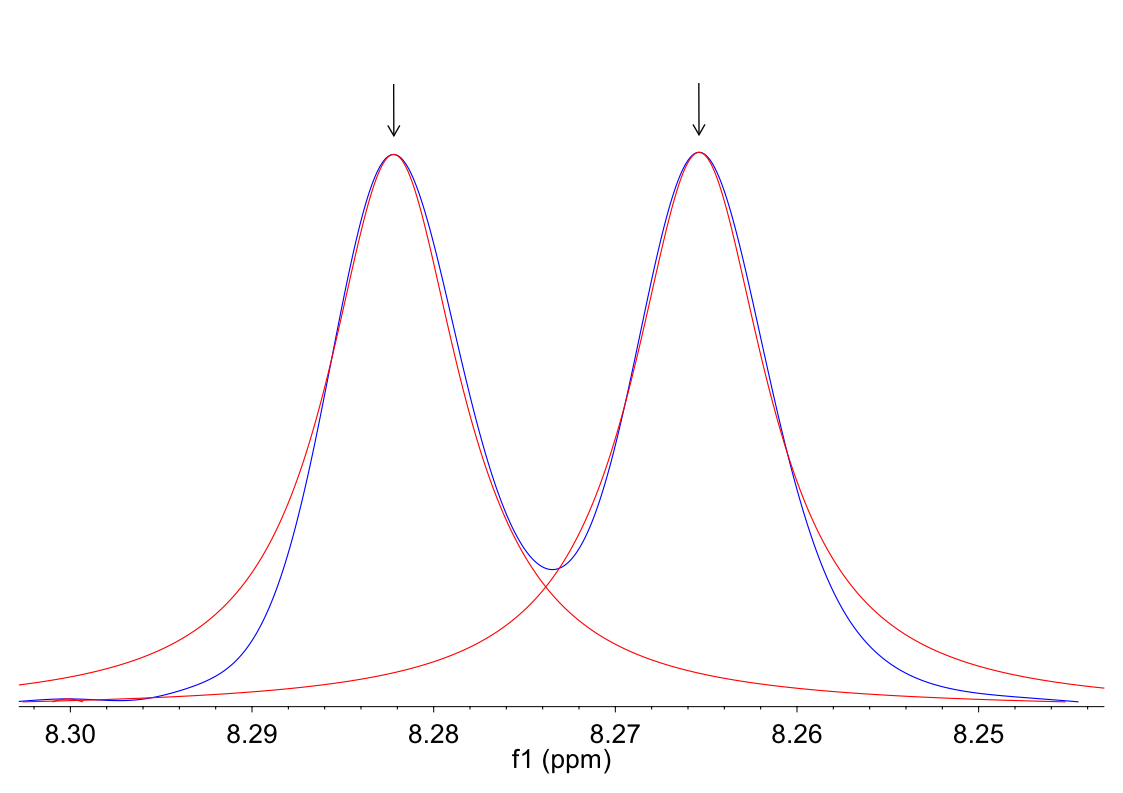 | 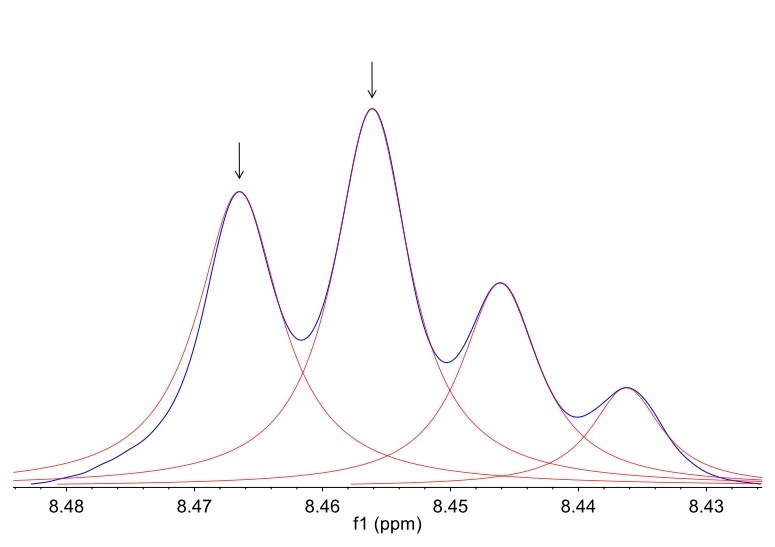 |
| GEP(pH=6) | GEG(pH=6) |

**Figure S2** Amide region of 1D NMR spectra for AcGXGNH_2_ and AcGXPNH_2_ peptides. Arrows indicates the splitting of corresponding amide signals for residue X. Blue spectra are the measured ones and red spectra are the resulting peaks from fittings.

| (a) | (b) |
| --- | --- |

**Figure S3** The correlation of ΔG(β to PII) derived for AcGXGNH_2_ and the β-sheet scale by Kim and Berg, in which (a) is the plot for non-polar amino acids (R = 0.89) and (b) for all amino acids with Asn and Phe excluded (R = 0.72).

***Effects of different ^3^J_αN_(PII) and ^3^J_αN_(β) values on data analysis***

Using our previous set of residue-specific ^3^J_αN_ reference values^1,2^ to analyze the data in this study, we obtain slightly different PII, β and α-contents for X in AcGXGNH_2_. We observe similar correlations between the new results and the β sheet scale by Kim and Berg^3,4^, and between the new results and the results for dipeptides^5^ (Figure S4 and S5; Figure S3 (a) *vs*. Figure S4 (a), R=0.89 *vs*. R=0.86; Figure S3 (b) *vs*. Figure S4 (b), R=0.72 *vs*. R=0.76; Figure S5 *vs*. Figure 3, R=0.81 *vs*. R=0.84, respectively, for comparison). It is obvious that all correlations are kept quite well, either we assign ^3^J_αN_ reference values to a set of residue-specific ^3^J_αN_ values, or to 5.42 and 9.30 Hz for PII and β conformations, respectively; the correlations derived with residue-specific ^3^J_αN_ reference values deteriorate slightly.

| (a) | (b) |
| --- | --- |

**Figure S4** The correlation of the β-sheet scale by Kim & Berg and ΔG(β to PII) derived for AcGXGNH_2_ using a set of residue-specific ^3^J_αN_ reference values for PII and β conformations, in which (a) is the plot for non-polar amino acids and (b) for all amino acids with His and Ser excluded.

**Figure S5** The correlation of ΔG(β to PII) derived for amino acid dipeptides and that for AcGXGNH_2_ using a set of residue-specific ^3^J_αN_ reference values for PII and β conformations (Gln, Cys and Asn labeled in red are outliers).

The free energy-conformation diagrams derived based on a set of residue-specific ^3^J_αN_ reference values^1,2^ are shown in Figure S6. To compare two sets of diagrams (Figure S6 and Figure 4), we have compared differences in free energy level of either two conformational basins (α and β, α and PII, β and PII) between two sets of diagrams for all amino acids. The results are shown in Figure S7, S8 and S9 for ΔG(α/β) (residue-specific *vs*. 5.42/9.30 Hz, R=0.99), ΔG(α/PII) (residue-specific *vs*. 5.42/9.30 Hz, R=0.99) and ΔG(β/PII) (residue-specific *vs*. 5.42/9.30 Hz, R=0.81), respectively. Strict comparison indicate that different ^3^J_αN_(PII) and ^3^J_αN_(β) values do have insignificant effects on specific amino acids (Asn, Val and Gln, for example). Nevertheless, two sets of results are matched to each other overall with derived conclusions being the same.

| 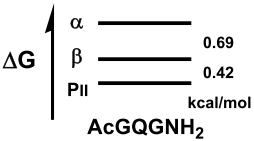 | 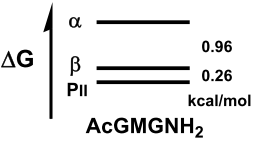 | 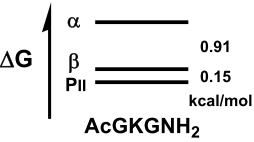 | 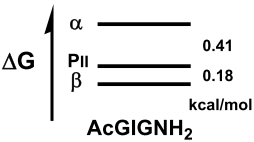 |
| --- | --- | --- | --- |
| 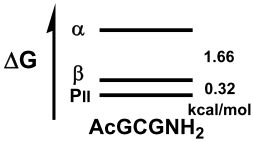 | 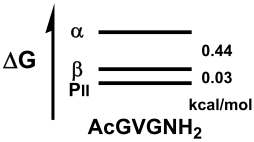 | 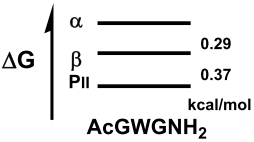 | 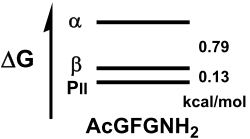 |
| 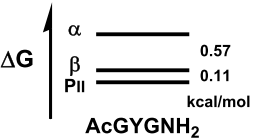 | 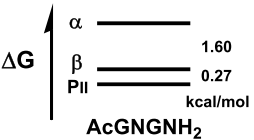 | 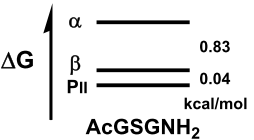 | 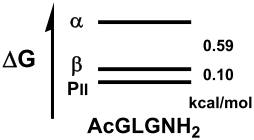 |
| 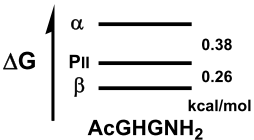 | 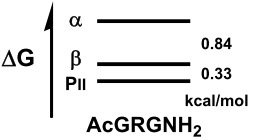 | 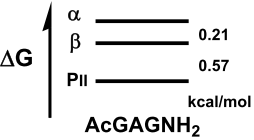 | 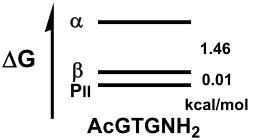 |
| 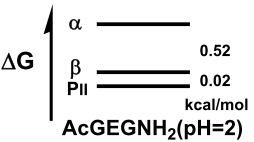 | 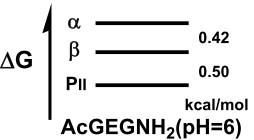 | 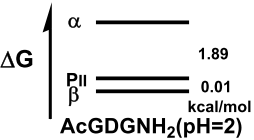 | 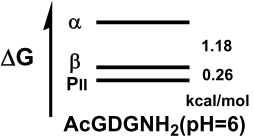 |

**Figure S6** Derived free energy-conformation diagrams for AcGXGNH_2_ using a set of residue-specific ^3^J_αN_ reference values for PII and β conformations.

**Figure S7** The correlation of ΔG(α to β) values derived for AcGXGNH_2_ using ^3^J_αN_ =5.42/9.30 Hz for PII and β conformations and those derived using a set of residue-specific ^3^J_αN_ reference values for PII and β conformations.

**Figure S8** The correlation of ΔG(α to PII) values derived for AcGXGNH_2_ using ^3^J_αN_ =5.42/9.30 Hz for PII and β conformations and those derived using a set of residue-specific ^3^J_αN_ reference values for PII and β conformations.

**Figure S9** The correlation of ΔG(β to PII) values derived for AcGXGNH_2_ using ^3^J_αN_ =5.42/9.30 Hz for PII and β conformations and those derived using a set of residue-specific ^3^J_αN_ reference values for PII and β conformations.

**Table S1** Derived α-contents of X in AcGXGNH_2_ and the relative rates of disulfide formation in a synthetic model for limited amino acids.

|  | x_α_ (%) in AcGXGNH_2_ | relative rates* |
| --- | --- | --- |
| Ala | 16.3% | 2.86 |
| Ile | 17.6% | 2.14 |
| Lys | 8.6% | 1.14 |
| Leu | 14.5% | 2.71 |
| Val | 18.7% | 2.86 |
| Asp | 5.0% | 0.86 |

* The relative rates of disulfide formation are those measured by Miller *et al*. ^7^

| GAG | 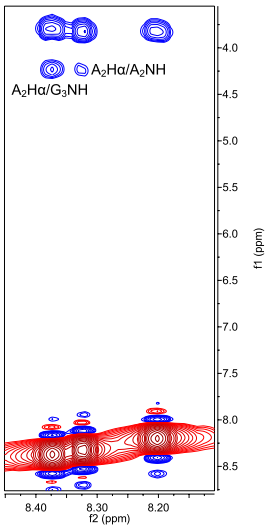  (a) | 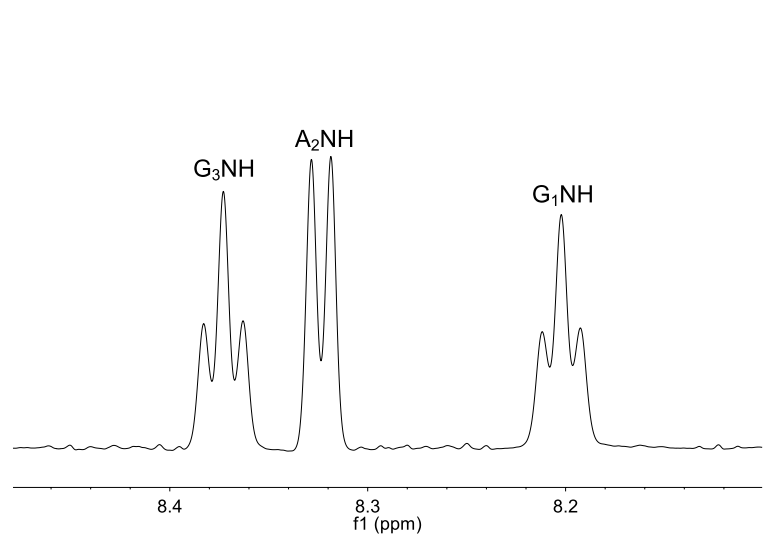(b) |  |
| --- | --- | --- | --- |
|  |  | 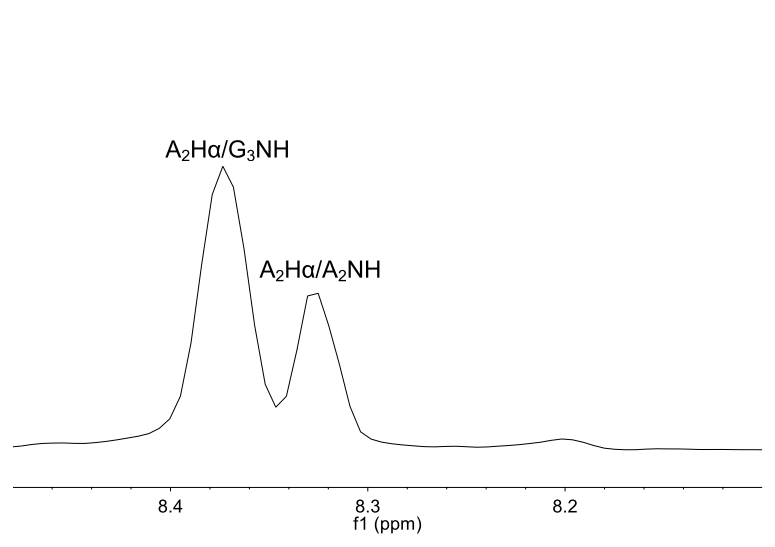(c) |  |
| GCG | 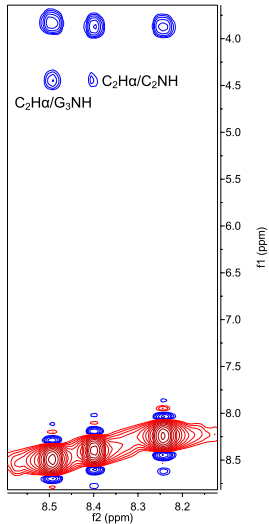  (a) | 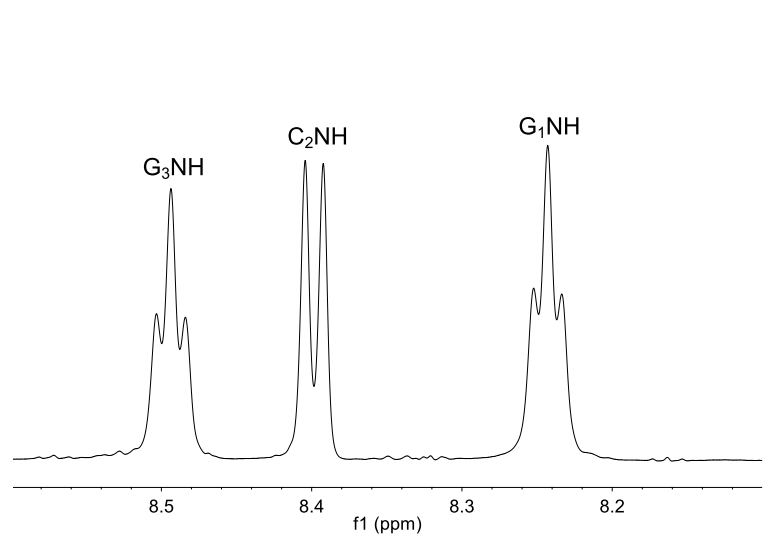(b) |  |
|  |  | 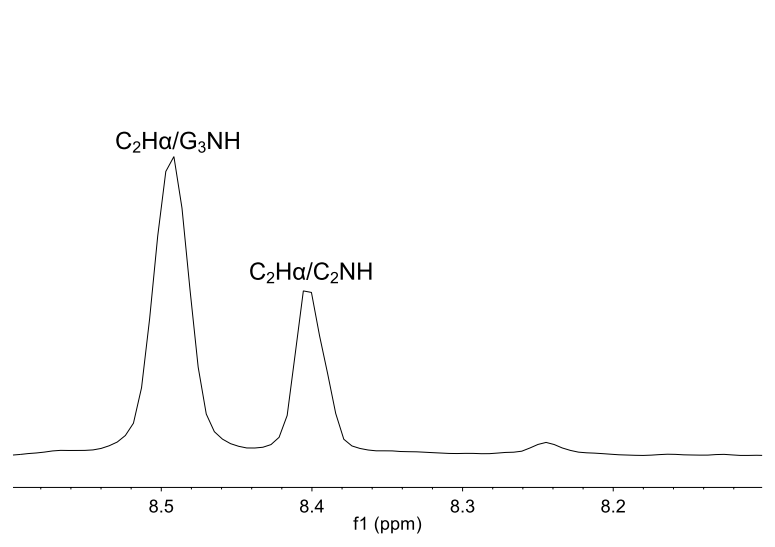(c) |  |
| GFG | 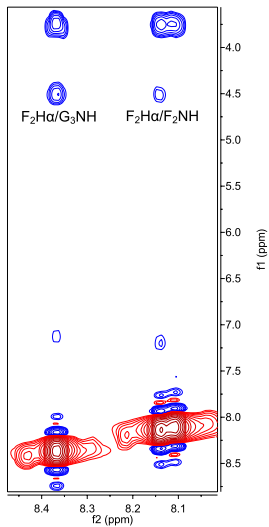  (a) | 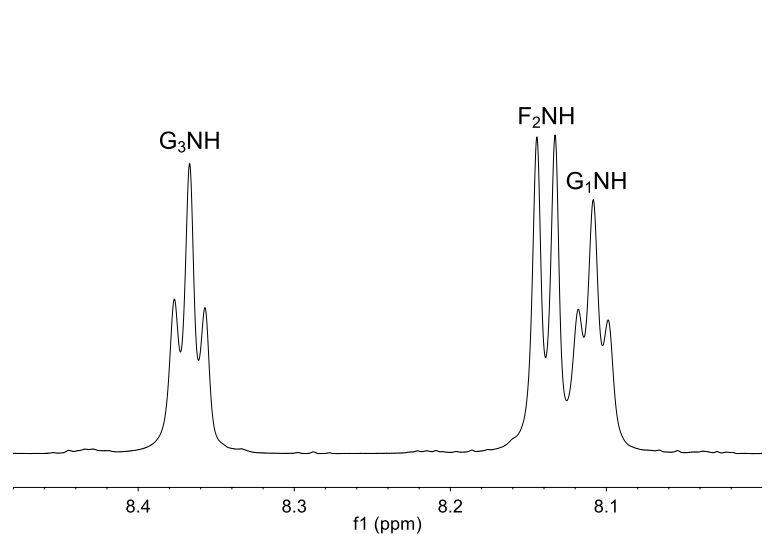 (b) |  |
|  |  | 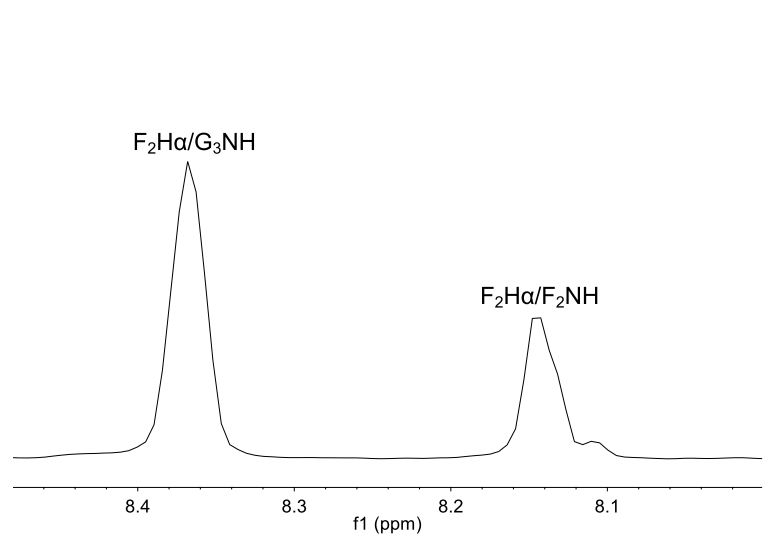 (c) |  |
| GHG | 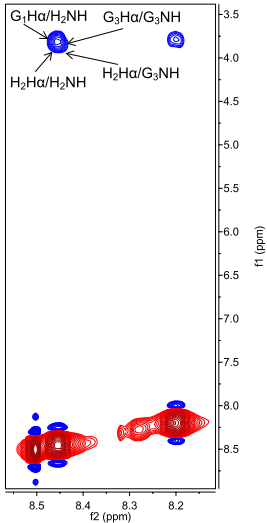  (a) | 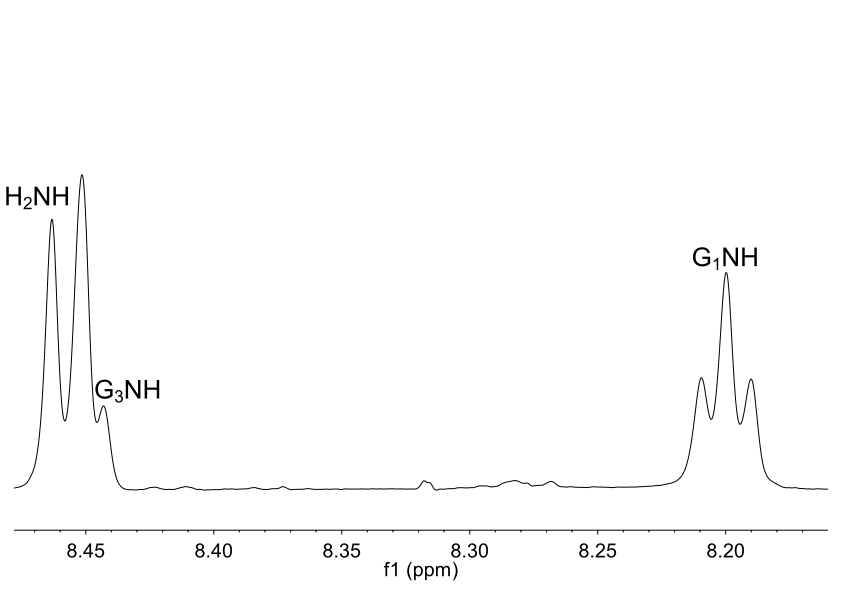 (b) |  |
|  |  | 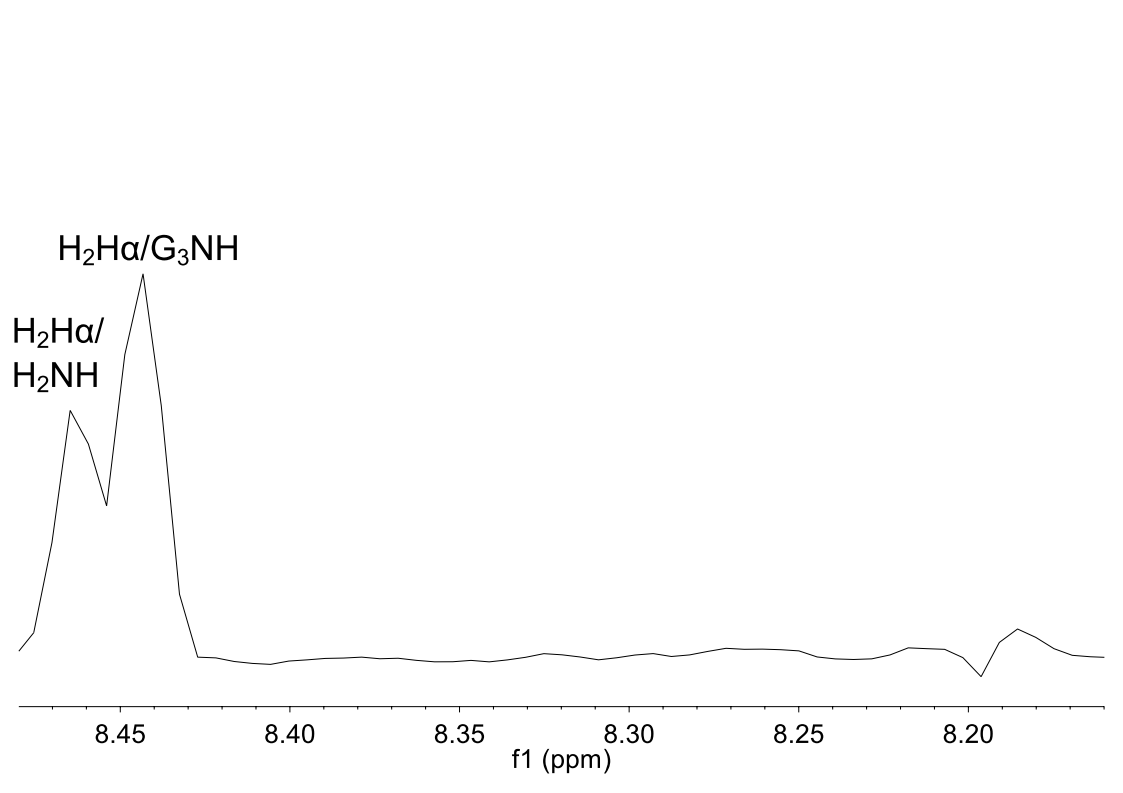 (c) |  |
| GIG | 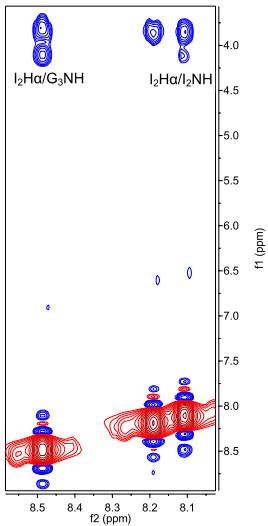  (a) | 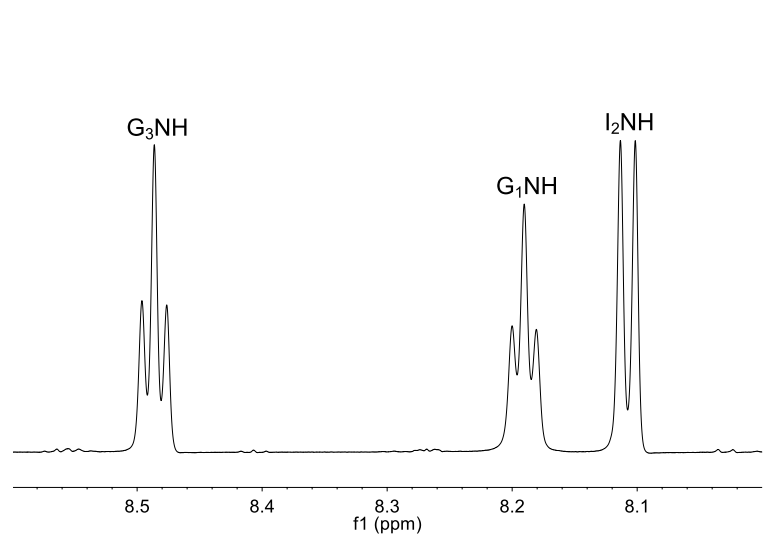 (b) |  |
|  |  | 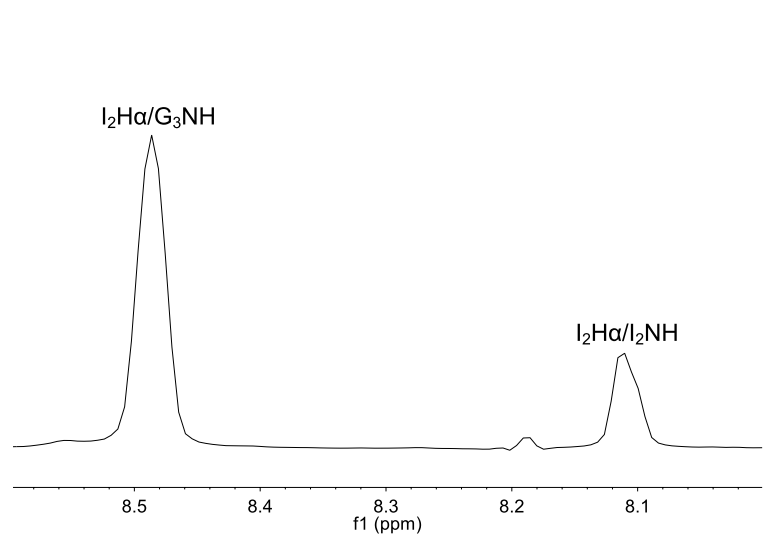 (c) |  |
| GKG | 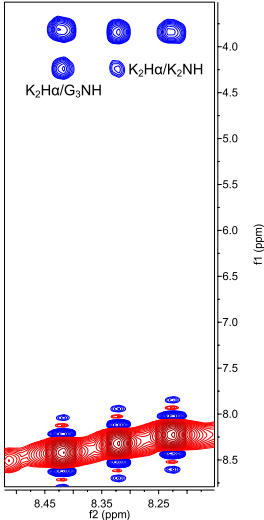  (a) | 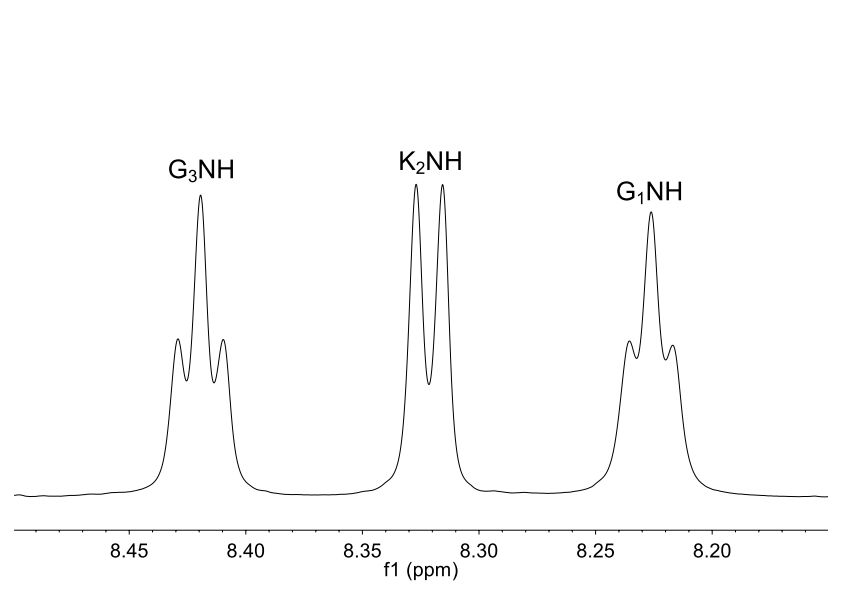 (b) |  |
|  |  | 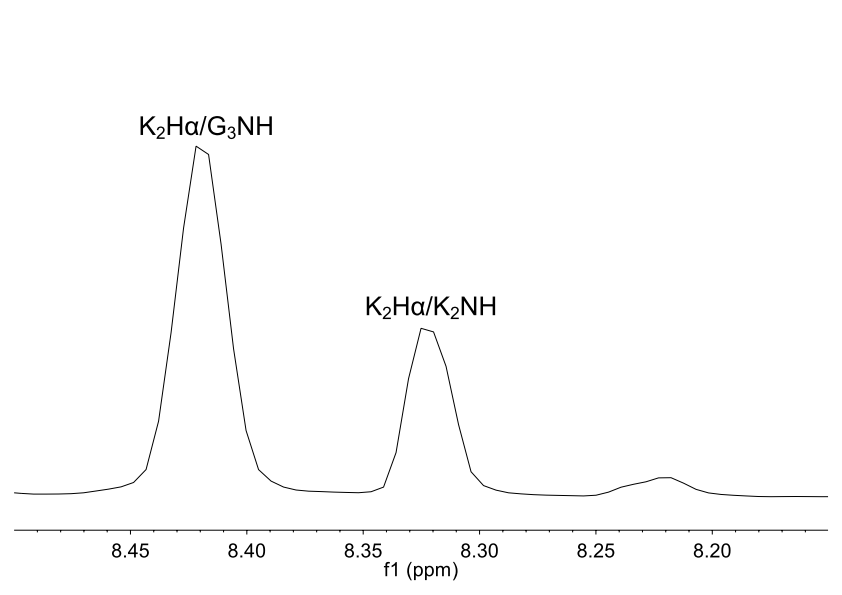 (c) |  |
| GLG | 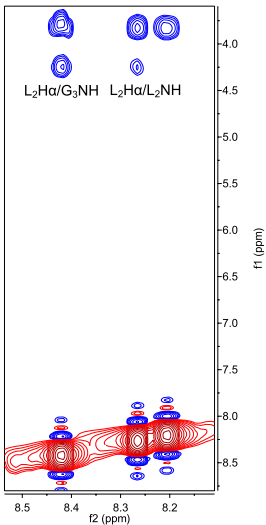  (a) | 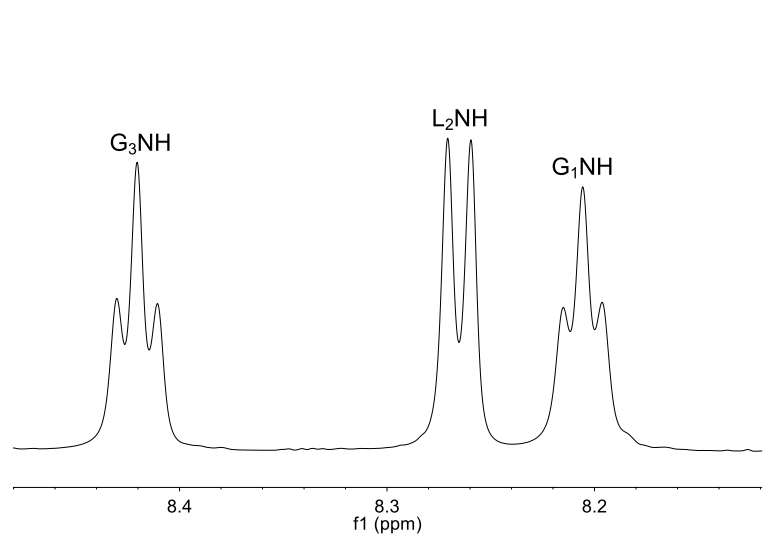(b) |  |
|  |  | (c) |  |
| GMG | (a) | (b) |  |
|  |  | (c) |  |
| GNG | (a) | (b) |  |
|  |  | (c) |  |
| GQG | (a) | (b) |  |
|  |  | (c) |  |
| GRG | (a) | (b) |  |
|  |  | (c) |  |
| GSG | (a) | (b) |  |
|  |  | (c) |  |
| GTG | (a) | (b) |  |
|  |  | (c) |  |
| GVG | (a) | (b) |  |
|  |  | (c) |  |
| GWG | (a) | (b) |  |
|  |  | (c) |  |
| GYG | (a) | (b) |  |
|  |  | (c) |  |
| GDG  pH=2 | (a) | (b) | |
|  |  | (c) | |
| GDG  pH=6 | (a) | (b) | |
|  |  | (c) | |
| GEG  pH=2 | (a) | (b) | |
|  |  | (c) | |
| GEG  pH=6 | (a) | (b) | |
|  |  | (c) | |

**Figure S10** Panels labelled with (a) show amide region of NOESY spectra for AcGXGNH_2_ peptides; Panels labelled with (b) & (c) compare the amide regions of 1D NMR spectra and the corresponding one-dimensional traces from NOESY spectra that indicates the relative intensities of d_αN_(i, i) and d_αN_(i, i + 1) NOEs.

**Table S2** Derived α, PII and β-contents for X in AcGXGNH_2_ with RR = 0.80, 0.85 and 0.90 (For comparison, see Table 1 for the corresponding values when RR = 1.00).

| Amino acids | RR = 0.80 | | | RR = 0.85 | | | RR = 0.90 | | |
| --- | --- | --- | --- | --- | --- | --- | --- | --- | --- |
|  | x_α_ (%) | x_PII_ (%) | x_β_ (%) | x_α_ (%) | x_PII_ (%) | x_β_ (%) | x_α_ (%) | x_PII_ (%) | x_β_ (%) |
| Tyr | 20.1% | 36.8% | 43.1% | 18.7% | 38.7% | 42.6% | 17.3% | 40.5% | 42.2% |
| Trp | 23.3% | 41.4% | 35.3% | 21.8% | 43.4% | 34.8% | 20.4% | 45.3% | 34.3% |
| Thr | 4.0%* | 36.4% | 59.6% | 4.0%* | 36.4% | 59.6% | 4.0%* | 36.4% | 59.6% |
| Arg | 14.0% | 41.7% | 44.3% | 12.4% | 43.8% | 43.8% | 10.9% | 45.8% | 43.3% |
| Gln | 15.2% | 40.3% | 44.5% | 13.7% | 42.3% | 44.0% | 12.2% | 44.3% | 43.5% |
| Asn | 2.5%* | 39.8% | 57.7% | 2.5%* | 39.8% | 57.7% | 2.5%* | 39.8% | 57.7% |
| Met | 13.2% | 40.8% | 46.0% | 11.6% | 42.9% | 45.5% | 10.1% | 44.9% | 45.0% |
| Leu | 20.0% | 37.4% | 42.6% | 18.6% | 39.3% | 42.1% | 17.2% | 41.1% | 41.7% |
| Lys | 14.6% | 41.8% | 43.6% | 13.0% | 43.9% | 43.1% | 11.5% | 45.9% | 42.6% |
| Ile | 21.5% | 24.3% | 54.2% | 20.5% | 25.6% | 53.9% | 19.5% | 27.0% | 53.5% |
| Cys | 8.3% | 41.6% | 50.1% | 6.8% | 43.7% | 49.5% | 5.2% | 45.8% | 49.0% |
| His | 20.9% | 23.8% | 55.3% | 19.9% | 25.1% | 55.0% | 19.0% | 26.4% | 54.6% |
| Ala | 21.8% | 59.5% | 18.7% | 20.3% | 61.5% | 18.2% | 18.9% | 63.4% | 17.7% |
| Phe | 16.0% | 37.4% | 46.6% | 14.5% | 39.3% | 46.2% | 13.1% | 41.2% | 45.7% |
| Ser | 16.5% | 40.8% | 42.7% | 15.0% | 42.8% | 42.2% | 13.5% | 44.8% | 41.7% |
| Val | 22.6% | 23.9% | 53.5% | 21.6% | 25.2% | 53.2% | 20.6% | 26.5% | 52.9% |
| Glu (pH=2) | 21.7% | 31.3% | 47.0% | 20.4% | 33.0% | 46.6% | 19.2% | 34.6% | 46.2% |
| Glu (pH=6) | 18.9% | 50.0% | 31.1% | 17.3% | 52.2% | 30.5% | 15.7% | 54.3% | 30.0% |
| Asp (pH=2) | 2.0%* | 40.2% | 57.8% | 2.0%* | 40.2% | 57.8% | 2.0%* | 40.2% | 57.8% |
| Asp (pH=6) | 5.0%* | 49.8% | 45.2% | 5.0%* | 49.8% | 45.2% | 5.0%* | 49.8% | 45.2% |

**Table S3** Derived α, PII and β-contents for X in AcGXGNH_2_ with RR = 0.95, 1.05 and 1.10 (For comparison, see Table 1 for the corresponding values when RR = 1.00).

| Amino acids | RR = 0.95 | | | RR = 1.05 | | | RR = 1.10 | | |
| --- | --- | --- | --- | --- | --- | --- | --- | --- | --- |
|  | x_α_ (%) | x_PII_ (%) | x_β_ (%) | x_α_ (%) | x_PII_ (%) | x_β_ (%) | x_α_ (%) | x_PII_ (%) | x_β_ (%) |
| Tyr | 16.0% | 42.3% | 41.7% | 13.4% | 45.7% | 40.9% | 12.2% | 47.4% | 40.4% |
| Trp | 19.0% | 47.2% | 33.8% | 16.3% | 50.8% | 32.9% | 15.0% | 52.5% | 32.5% |
| Thr | 4.0%* | 36.4% | 59.6% | 4.0%* | 36.4% | 59.6% | 4.0%* | 36.4% | 59.6% |
| Arg | 9.4% | 47.8% | 42.8% | 6.6% | 51.6% | 41.8% | 5.2% | 53.4% | 41.4% |
| Gln | 10.7% | 46.3% | 43.0% | 7.9% | 50.0% | 42.1% | 6.6% | 51.8% | 41.6% |
| Asn | 2.5%* | 39.8% | 57.7% | 2.5%* | 39.8% | 57.7% | 2.5%* | 39.8% | 57.7% |
| Met | 8.6% | 46.9% | 44.5% | 5.8% | 50.7% | 43.5% | 4.4% | 52.5% | 43.1% |
| Leu | 15.9% | 42.9% | 41.2% | 13.2% | 46.5% | 40.3% | 12.0% | 48.1% | 39.9% |
| Lys | 10.0% | 47.9% | 42.1% | 7.2% | 51.7% | 41.1% | 5.8% | 53.6% | 40.6% |
| Ile | 18.5% | 28.3% | 53.2% | 16.6% | 30.9% | 52.5% | 15.6% | 32.2% | 52.2% |
| Cys | 3.7% | 47.8% | 48.5% | 0.7% | 51.8% | 47.5% | 0% | 53.3% | 46.7% |
| His | 18.0% | 27.7% | 54.3% | 16.1% | 30.2% | 53.7% | 15.1% | 31.5% | 53.4% |
| Ala | 17.5% | 65.2% | 17.3% | 15.0% | 68.6% | 16.4% | 13.9% | 70.1% | 16.0% |
| Phe | 11.8% | 43.0% | 45.2% | 9.1% | 46.6% | 44.3% | 7.8% | 48.3% | 43.9% |
| Ser | 12.1% | 46.7% | 41.2% | 9.3% | 50.5% | 40.2% | 7.9% | 52.3% | 39.8% |
| Val | 19.7% | 27.8% | 52.5% | 17.8% | 30.3% | 51.9% | 16.8% | 31.6% | 51.6% |
| Glu (pH=2) | 18.0% | 36.2% | 45.8% | 15.7% | 39.4% | 44.9% | 14.6% | 40.9% | 44.5% |
| Glu (pH=6) | 14.2% | 56.3% | 29.5% | 11.3% | 60.2% | 28.5% | 10.0% | 62.0% | 28.0% |
| Asp (pH=2) | 2.0%* | 40.2% | 57.8% | 2.0%* | 40.2% | 57.8% | 2.0%* | 40.2% | 57.8% |
| Asp (pH=6) | 5.0%* | 49.8% | 45.2% | 5.0%* | 49.8% | 45.2% | 5.0%* | 49.8% | 45.2% |

|  | |
| --- | --- |
|  |  |
|  |  |
|  |  |

**Figure S11** Comparison of the correlations between determined α-contents for AcGXGNH_2_ and the relative rates of disulfide formation in a synthetic model (RR = 0.80 - 1.10).

|  | |
| --- | --- |
|  |  |
|  |  |
|  |  |

**Figure S12** Comparison of the correlations between ΔG(β to PII) derived for AcGXGNH_2_ and the β-sheet scale by Kim and Berg for all amino acids with Asn and Phe excluded (RR = 0.80 - 1.10).

|  | |
| --- | --- |
|  |  |
|  |  |
|  |  |

**Figure S13** Comparison of the correlations between ΔG(β to PII) derived for AcGXGNH_2_ and that for dipeptides (RR = 0.80 - 1.10).

**Material and Methods**

***Derivation of Equation (1)***

For AcGXPNH_2_, equations (a) and (b) apply; for AcGXGNH_2_, equations (c) and (d) apply. Assuming the PII to β population ratio of X in AcGXPNH_2_ and AcGXGNH_2_ is approximately the same, that is, x_PII_(GXG)/ x_β_(GXG) = x_PII_(GXP)/ x_β_(GXP), then x_PII_(GXG)/ [ x_PII_(GXG)+x_β_(GXG) ] = x_PII_(GXP)/ [ x_PII_(GXP)+x_β_(GXP) ] and we get equation (e) after substituting equation (b) into it. From equations (c) and (e), we can derive equation (f); similarly x_β_ (GXG)/ [ x_PII_(GXG)+x_β_(GXG) ] = x_β_ (GXP)/ [ x_PII_(GXP)+x_β_(GXP) ] = x_β_ (GXP), we can derive equation (g). Substituting equations (f) and (g) into equation (d), we get equation (h); in the equation (h), we can substitute ^3^J_αN_(PII) • x_PII_(GXP) + ^3^J_αN_(β) • x_β_(GXP) with ^3^J_αN_(GXP), that is, equation (a), and rearrange the equation, then we get equation (i). From equation (i), we can derive the equation (1).

^3^J_αN_(PII) • x_PII_(GXP) + ^3^J_αN_(β) • x_β_(GXP) = ^3^J_αN_(GXP) (a)

x_PII_(GXP) + x_β_(GXP) = 1 (b)

x_PII_(GXG) + x_β_(GXG) + x_α_(GXG) = 1 (c)

^3^J_αN_(PII) • x_PII_(GXG) + ^3^J_αN_(β) • x_β_(GXG) + ^3^J_αN_(α) • x_α_(GXG) = ^3^J_αN_(GXG) (d)

x_PII_(GXG)/ [ x_PII_(GXG)+x_β_(GXG) ] = x_PII_(GXP) (e)

x_PII_(GXG) = x_PII_(GXP) • [ x_PII_(GXG)+x_β_(GXG) ] = x_PII_(GXP) • [ (1 - x_α_(GXG) ] (f)

x_β_ (GXG) = x_β_ (GXP) • [ x_PII_(GXG)+x_β_(GXG) ] = x_β_ (GXP) • [ (1 - x_α_(GXG) ] (g)

^3^J_αN_(PII) • x_PII_(GXP) • [ (1 - x_α_(GXG) ] + ^3^J_αN_(β) • x_β_ (GXP) • [ (1 - x_α_(GXG) ]

+ ^3^J_αN_(α) • x_α_(GXG) = ^3^J_αN_(GXG) (h)

^3^J_αN_(GXP) - ^3^J_αN_(GXG) = ^3^J_αN_(GXP) • x_α_(GXG) - ^3^J_αN_(α) • x_α_(GXG) (i)

x_α_(GXG) = [ ^3^J_αN_(GXP) - ^3^J_αN_(GXG)]/[ ^3^J_αN_(GXP) - ^3^J_αN_(α)] (1)

***Peptide Synthesis and purification***

Peptides were assembled on Rink Amide resin (GL Biochem Ltd., Shanghai China ) with an automated peptide synthesizer Focus XC with 6 channels (Advanced Automated Protein Technologies, Louisville, KY, USA) by using 9-fluorenylmethyloxylcarbonyl (Fmoc) chemistry. Fmoc-amino acids, O-(Benzotriazol-1-yl)-N,N,N’,N’-tetramethyluronium tetrafluoroborate (TBTU) and N-hydroxybenzotriazole (HOBT) were purchased from GL Biochem Ltd., Shanghai China. The N terminus of each peptide is capped with acetic anhydride after assembly on the solid matrix. Cleavage of peptides from the resin was routinely performed using 95% trifluoroacetic acid (TFA) in the presence of the scavenger 2.5% triisopropylsilane (TIS) and 2.5% H_2_O. The products were precipitated with cold ether. Water-soluble peptides were lyophilized overnight and directly purified on a reverse-phase HPLC using a C-18 semi-preparative column (Amethyst C18-H, 10.0 x 250 mm, Sepax Techlogies, Inc., Newark, Delaware, USA) with water and acetonitrile in gradients (0.1% TFA in both phases). Fractions containing the product were pooled and lyophilized. The identity and molecular weight of each peptide were confirmed by ^1^H NMR and matrix-assisted laser desorption/ionization time-of-flight (MALDI-TOF) mass spectrometry.

***CD measurements***

Circular dichroism (CD) spectra were recorded on a J-810 spectrometer (JASCO, Japan) using 0.1-cm path-length quartz CD cuvettes. The corresponding solvent CD background was measured and subtracted from the sample spectrum. Wavelength scans were performed from 260 nm to 190 nm at 25°C. CD measurements were carried out with about 100 μM - 500 μM peptides in 10 mM phosphate with pH adjust to 4.0 (pH = 2.0 and 6.0 for Asp and Glu). Spectra were collected with a 0.5 or 1 nm resolution and a scan rate of 1 nm s^−1^. Temperature was maintained and controlled with a temperature control system (**JULABO F-12)**. Reported spectra are averages of 12 or more scans. Each spectrum was measured at least 3 times with individually prepared peptide solutions and expressed as molar ellipticity. The concentrations of peptides were determined from a combination of UV absorbance and NMR peak integration. In this procedure, a CD sample solution of unknown concentration was mixed with a small but known amount of tryptophan stock solution. A 1D proton NMR spectrum with water suppression was then recorded on the mixture. The non exchangeable β protons of Trp (around 3.2 ppm) and those of the corresponding protons of the acetyl group on the N-terminus of each peptide (around 2.0 ppm) or the H_β_ from the side chain of the non-glycine residue, were then integrated to give a quantitative ratio, from which the peptide concentration could be calculated from the concentration of tryptophan as determined from its UV absorbance at 279 nm.

***NMR spectroscopy***

NMR samples were made with 1-5 mM peptides in 25 mM phosphate buffer (10% D_2_O) with pH adjust to 4.0 (pH = 2.0 and 6.0 for Asp and Glu). NMR measurements were carried out on Bruker AVANCE 400/600 MHz spectrometers at 25°C. The phase-sensitive total correlation spectroscopy (TOCSY) experiments were carried out with a mixing time of 80 ms. NOESY experiments were carried out with mixing times of 400 ms. Each two-dimensional data set contained 256 FIDs with 2048 complex data points each, obtained by collecting 32 or 64 added free induction decays after four dummy scans. Spectra were Fourier transformed in both t2 and t1 dimensions after apodization with a shifted square sine bell function, typically with 90° phase shift. Zero filling was done in the t1 dimension to obtain a final matrix of 2048 x 1024 real points. ^3^J_αN_ coupling constants were determined from high resolution 1D spectra recorded with 64 or more scans using an acquisition time of 4.4 s and a sweep width of 7200 Hz. Water suppression was achieved using a 3919 Watergate sequence^6^. The original free induction decays were zero-filled to 128K data points and Fourier transformed with exponential window function applied. The ^3^J_αN_ constants were measured directly by the splitting of amide proton signals through a peak-fitting procedure to Lorentzian line shape. The accuracy of coupling constants is believed to be within 0.05 Hz for non-overlapping peaks.

**References:**

1. Shi, Z. et al. Polyproline II propensities from GGXGG peptides reveal an anticorrelation with β-sheet scales. *Proc. Natl. Acad. Sci. U.S.A.* **102**, 17964-17968 (2005).

2. Avbelj, F. & Baldwin, R.L. Role of backbone solvation and electrostatics in generating preferred peptide backbone conformations: distributions of phi. *Proc. Natl. Acad. Sci. U.S.A.* **100**, 5742-5747 (2003).

3. Kim, C.A. & Berg, J.M. Thermodynamic beta-sheet propensities measured using a zinc-finger host peptide. *Nature* **362**, 267-270 (1993).

4. Smith, C.K., Withka, J.M. & Regan, L. A thermodynamic scale for the β-sheet forming tendencies of the amino acids. *Biochemistry* **33**, 5510-5517 (1994).

5. Grdadolnik, J., Mohacek-Grosev, V., Baldwin, R.L. & Avbelj, F. Populations of the three major backbone conformations in 19 amino acid dipeptides. *Proc. Natl. Acad. Sci. U.S.A.* **108**, 1794-1798 (2011).

6. Piotto, M., Saudek, V. & Sklenář, V. Gradient-tailored excitation for single-quantum NMR spectroscopy of aqueous solutions. *J. Biomol. NMR* **2**, 661-665 (1992).

7. Miller, S.E., Watkins, A.M., Kallenbach, N.R. & Arora, P.S. Effects of side chains in helix nucleation differ from helix propagation. *Proc. Natl. Acad. Sci. U.S.A.* **111**, 6636-6641 (2014).
